# The effects of mindfulness-based interventions in medical students: a systematic review

**DOI:** 10.1007/s10459-023-10231-0

**Published:** 2023-05-25

**Authors:** Ilona Kaisti, Petri Kulmala, Mirka Hintsanen, Tuula Hurtig, Saara Repo, Tiina Paunio, Jouko Miettunen, Anu-Helmi Halt, Erika Jääskeläinen

**Affiliations:** 1 Research Unit of Population Health, University of Oulu, Oulu, Finland; 2 Faculty of Medicine, University of Oulu, Oulu, Finland; 3 Department of Pediatrics, Oulu University Hospital, University of Oulu, Oulu, Finland; 4 Medical Research Center, Oulu University Hospital, University of Oulu, Oulu, Finland; 5 The Faculty of Education and Psychology, University of Oulu, Oulu, Finland; 6 Research Unit of Clinical Medicine, Psychiatry, Child Psychiatry, University of Oulu, Oulu, Finland; 7 Clinic of Child Psychiatry, Oulu University Hospital, University of Oulu, Oulu, Finland; 8 Clinicum, Faculty of Medicine, University of Helsinki, Helsinki, Finland; 9 Department of Psychiatry and SleepWell Research Program, Faculty of Medicine, Helsinki University Central Hospital, University of Helsinki, Helsinki, Finland; 10 Medical Research Center Oulu, Oulu University Hospital, University of Oulu, Oulu, Finland; 11 Research Unit of Clinical Medicine, Psychiatry, University of Oulu, Oulu, Finland; 12 Department of Psychiatry, Oulu University Hospital, University of Oulu, Oulu, Finland

**Keywords:** Mindfulness, Medical student, Intervention, Mental health

## Abstract

The number of studies on the effects of mindfulness on healthcare professionals is increasing. The main aim of this study was to collate the quantitative results of original studies analyzing the effects of mindfulness-based interventions on a variety of outcomes in medical students. We also analyzed how the study design and characteristics of the intervention affect the results, and identified qualitative effects of mindfulness interventions. A literature search was performed in different databases in June 2020. Original articles meeting the following criteria were included: (1) at least 50% of the participants were medical students, (2) included a mindfulness intervention, (3) analyzed any outcome relating to mindfulness intervention, (4) peer-reviewed (5) written in English. Eventually, 31 articles including 24 different samples were included. Over half of the studies were RCTs. In over half of the studies, the intervention was 4- to 10-week original Mindfulness-Based Stress Reduction or Mindfulness-Based Cognitive Therapy or a modification of these. In general, satisfaction with the interventions was good. Based on a meta-analysis, after the intervention, the intervention group had statistically significantly fewer symptoms of stress and distress and had higher mindfulness than the controls. The beneficial effects persisted in follow-ups over months or years. Both long and shorter courses and courses with and without face-to-face sessions were effective. Both controlled and uncontrolled studies had statistically significant results. Qualitative results revealed potential factors behind the quantitative effects. The number of studies on mindfulness interventions in medical students has increased drastically. Mindfulness-based interventions seem to offer a good possibility to enhance medical students’ well-being.

**Supplementary Information:**

The online version contains supplementary material available at 10.1007/s10459-023-10231-0.

## Introduction

Mindfulness means active awareness of the present moment without judging or the need to change things (Ludwig & Kabat-Zinn, 2008). Traditionally, mindfulness is based on Buddhist practice, but mindfulness itself does not contain religious or cultural beliefs. Instead, mindfulness focuses on awareness of the here and now and disengaging from intense attachment to thoughts, emotions, or beliefs (Ludwig & Kabat-Zinn, 2008). Mindfulness can be divided into state (i.e., a momentary condition) and trait (i.e., a permanent characteristic) mindfulness (Quaglia et al., 2016; Tomlinson et al., 2018). Trait mindfulness, also known as dispositional mindfulness, is associated positively with psychological health. Both state and trait mindfulness can be trained and increased by different mindfulness interventions (Quaglia et al., 2016; Tomlinson et al., 2018).

There are several structured mindfulness interventions. Probably the most well-known mindfulness intervention is the 8-week mindfulness-based stress reduction (MBSR) course originally developed by Jon Kabat-Zinn for the management of chronic pain (Kabat-Zinn et al., 1985). Another well-known mindfulness intervention is mindfulness-based cognitive therapy (MBCT) which was originally aimed at depression treatment (Creswell, 2017). In addition, several shorter mindfulness-based courses as well as internet and smartphone application mindfulness programs have been developed (Creswell, 2017).

A major increase in the number of publications on mindfulness can be seen especially in the last five years. The effectiveness of mindfulness has been researched in a variety of illnesses such as mental disorders, chronic pain, cancer, and gastrointestinal conditions (Tran et al., 2020). Mindfulness-based interventions are effective (equivalent to evidence-based, superior to other comparisons) in treating different psychiatric disorders such as depression and addictions (Goldberg et al., 2018). Mindfulness interventions are effective in decreasing stress and symptoms of depression and fatigue among breast cancer patients (Chang et al., 2021). In addition, mindfulness-based interventions can improve functioning and well-being and reduce the perception of pain and psychological symptoms in several chronic pain disorders (Hilton et al., 2017; Kabat-Zinn et al., 1985; Majeed et al., 2018).

During their studies, medical students go through many challenges such as facing death, diseases, and abusive patients. In addition, medical students learn to take responsibility and make important decisions. Several studies have indeed found that medical students around the world suffer from mental health problems, often more than their age-matched peers (Dyrbye et al., 2006; Hope et al., 2014; Puthran et al., 2016). Even though medical student well-being probably varies greatly between different countries, high demands, stress, and risk of mental disorders are common, and medical schools should offer support for medical students’ mental health.

Several studies have found evidence that mindfulness-based interventions could improve medical student well-being and have the potential to be adapted to different curricula (Daya & Hearn, 2018; McConville et al., 2017). McConville et al. (2017) made a systematic review of the effectiveness of mindfulness interventions in randomized and non-randomized controlled trials in health profession students, including 10 studies on medical students. They only included studies with quantitative outcomes. They found that mindfulness-based interventions statistically significantly decrease stress, anxiety, and depression and improve mindfulness and mood in health profession students when compared to controls who in most studies were passive controls. The results were not presented separately for medical students. Daya and Hearn (2018) made a quantitative systematic review of 12 studies on the effects of mindfulness on medical students’ stress, depression, fatigue, and burnout. Most of the studies included a control group, usually a passive control group. Based on their results, stress and depression decreased statistically significantly. They included diverse mindfulness interventions, such as well-being interventions which had mindfulness as part of the intervention.

Original studies on mindfulness interventions on medical students have been published in recent years, after reviews by McConville et al. (2017) and Daya and Hearn (2018). However, to our knowledge, there is no systematic review collating the effects of all MBSR and other structured mindfulness programs specifically in the medical student population, including both quantitative and qualitative outcomes. This kind of systematic review is needed to understand the effects of mindfulness interventions on medical students’ health and to develop well-being interventions and curricula.

The main aim of this study was to collate and summarize the quantitative results of all the original studies analyzing the effects of mindfulness-based interventions on a variety of outcomes, for example, outcomes related to psychological functioning and well-being in medical students. We also aimed to analyze how the content and length, number and length of sessions and home practice, obligatoriness of the course, and other characteristics of the intervention affect the results of the intervention. As a secondary aim, based on qualitative data, we aimed to identify the qualitative effects of mindfulness interventions and potential factors behind the quantitative effects of mindfulness interventions.

## Methods

In our research process, we followed the PRISMA guideline. PRISMA has widely used, evidence-based tools for standardizing systematic reviews (Page et al., 2021). The PRISMA checklist is presented in Online Supplement Tables S1a and b. In addition, for studies including qualitative data, the ENTREQ statement (Tong et al., 2012) was followed (Online supplement Table S1c). There was no protocol of the review published.

### Literature search

The pre-planned literature search in five different databases was conducted on June 15, 2020, with the help of an informatician. The search covered Scopus, PubMed, Web of Science, Cinahl (EBSCO), PsycArticles (EBSCO), and Education Collection (ProQuest). The search phrase consisted of the terms “mindfulness”, “medical” and “student” (see Online Supplement Table S2 for the exact search algorithms). In addition, the search was limited to original articles that were peer-reviewed and written in English. Finally, a manual search was conducted from previous review articles and the references of some original articles.

### Inclusion and exclusion criteria

We included original articles meeting the following inclusion criteria: (1) at least 50% of the participants were medical students or the results were presented separately for the subpopulation of medical students, (2) included a mindfulness intervention consisting mainly of mindfulness elements (described in more detail below), (3) analyzed any outcome during or after mindfulness intervention (both quantitative and qualitative outcomes were included), and (4) original article published in a peer-reviewed publication (5) written in English.

Case reports and review articles were excluded. Studies with only one mindfulness session were also excluded.

We did not have any restrictions regarding sample size. The studies did not have to include a control group since this exclusion criterion would have decreased the number of studies notably.

We included studies with a variety of outcomes since we wanted to get a comprehensive view on the effects of mindfulness interventions in medical students (e.g., the feasibility of the intervention, all changes in physical and mental health, and participants’ experience of mindfulness interventions).

The studies had to include a structured mindfulness intervention including the formal practice of mindfulness meditation. In practice, studies using MBSR, modification of MBSR, or studies with similar intervention contents as in MBSR (i.e., meditation and theory about mindfulness) were included. The included interventions had to have mindfulness meditation as a core component, including home meditation practices. Interventions combining mindfulness with other intervention modalities were included, but the intervention had to focus on mindfulness. Interventions delivered face to face, via internet, mobile application, CD, or DVD were included. There was no requirement for the length of the mindfulness intervention, but interventions including only one session were excluded.

### Evaluation of the articles and data extraction

The abstracts of the search results were assessed separately by two authors (IK and EJ). In case of disagreement, the authors solved it through discussion.

The selected full texts were read and the following information was collected from each article (by IK): (1) basic information (article name, authors, year of publication, country, study design), (2) sample (group sizes, number of women/men, drop-outs, inclusion/exclusion criteria, age, year of medical school, ethnic background), (3) outcomes (type of outcome, measure/scale, time of data collection, length of follow-up, and results: changes from pre-test to post-test, from controlled studies differences between the intervention and comparison group after the intervention, persistence of changes during potential follow-up, and statistical significance of the result), and (4) characteristics of the mindfulness intervention (program content, student recruitment, duration, number and length of sessions, information on home practice, group size per session, obligatoriness, online material, program leader, time of day/year/studies).

In addition, regarding qualitative data, all text in the Results and Discussion of included articles with qualitative data were extracted electronically and entered into NVivo software (QSR International Pty Ltd. & released in March, 2020) by EJ.

### Quality assessment

For assessing the quality of quantitative studies, the Medical Education Research Study Quality Instrument (MERSQI) was applied (Cook & Reed, 2015). The MERSQI was chosen to be able to compare the quality of mindfulness studies to other studies in the field of Medical Education. For assessing the quality of qualitative studies, JBI’s Checklist for Qualitative Research was utilized (Lockwood et al., 2015). If the study was utilizing both quantitative and qualitative methods, both MERSQI and JBI’s Checklist for Qualitative Research were applied. The rationale for appraisal of the study was to assess the conduct and validity of the study. The quality rating of each study was performed by EJ and is presented in Supplemental Digital Tables S4a and b.

### Quantitative analyses

The quantitative results of the original studies were synthesized systematically. For studies including a control group and reporting all the needed values (sample size, mean and standard deviation of outcome for both intervention and control group) meta-analysis was performed with SPSS (IBM SPSS statistics version 28.0.1.1). A minimum of three original studies was required for performing a meta-analysis for an outcome. Based on the expected heterogeneity of the results, the random effects model was used to pool overall estimates of effect sizes (standardized mean differences between the groups, i.e. Cohen’s d). The effect sizes can be interpreted as d values 0.2 to 0.5 indicating a small effect; 0.5 to 0.8, medium effect; and 0.8 or more, a large effect (Cohen, 1992).

The heterogeneity of the studies was analyzed using the I^2^ statistic. Values of I^2^ range from 0 to 100%, reflecting the proportion of the total variation across studies beyond chance. A value of 25% indicates low, 50% moderate, and 75% high heterogeneity or major excessive variation across studies (Higgins et al., 2003). Possible publication bias was studied using Egger’s test for small-study effects.

An alpha level of 0.05 was used for all statistical tests.

### Qualitative analyses

Qualitative data was analyzed following thematic synthesis (Thomas & Harden, 2008). This method was chosen to construct themes that may explain the quantitative effects or lack of effects of mindfulness interventions. The process of deriving the themes was inductive. EJ assessed studies including qualitative data and did line-by-line coding on results and discussion sections of the included studies. During the process, subsequent studies were coded into pre-existing concepts, and new concepts were created when deemed necessary. Based on this, 55 codes were identified. These codes were then organized into 16 descriptive themes based on the content of the codes, the discussion between EJ and IK, and the authors’ knowledge of mindfulness interventions. Finally, analytical themes raised from the data were identified. During the process, EJ and IK discussed the found codes and arising themes. EJ has knowledge and experience of mindfulness interventions as a mindfulness teacher since 2019 (MBSR, and Compassionate Mindful Resilience (CMR) teacher), and IK is a medical student who has participated in 8 weeks MBSR course.

## Results

**Fig. 1:**
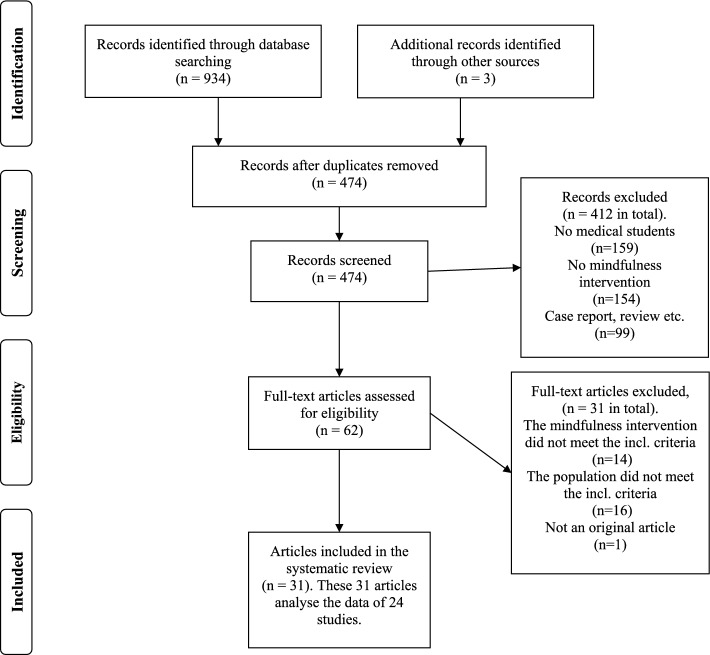
Flow diagram of identified, screened, included and excluded records

The searches produced 934 results. After the removal of duplicates, 471 publications were identified. In addition, three articles were found by manual search. After the evaluation of abstracts altogether 62 articles were selected for comprehensive evaluation. Eventually, 31 articles including 24 different study samples met our inclusion criteria and were included in this study (Fig. 1).

Most of the studies included quantitative results (23 studies), 5 were mixed including both quantitative and qualitative results, and one study was qualitative.

### Characteristics of the sample

All the included studies are described in Supplemental Digital Table S3.

Of all the studies included, one was published in the 1990s, one in 2000–2010, nine in 2010–2015, and 13 after 2015. The samples were from the US (n = 8), Canada (n = 3), Europe (n = 5), Australasia (n = 3), Brazil (n = 1) and Malaysia (n = 4). Regarding study design, 13 of the studies were randomized controlled trials, two were non-randomized controlled studies, eight were non-randomized pre-post-test studies (including 5 studies using mixed methods) without a control group, and one was a solely qualitative study without control group. The sample sizes varied. The number of subjects was under 50 in six studies, 50–99 in ten studies, 100–199 in four studies, and over 200 in four studies. The smallest sample size was 30 and the highest was 302.

In the included studies, the samples had a mean of 67% females (range = 46–84%); three studies did not report the gender distribution. The mean of all mean ages was 23 years (range 19–27 years) while seven studies did not report the age of the participants. In eight studies, the phase of studies was 1st–2nd year of medical school, in seven studies, 3rd–6th year of medical school, and in nine studies, there was a combination of students from the previous two groups. The way of reporting the phase of medical school varied between the articles due to differences in the curriculum structures in different countries. The outcomes in the studies assessed different aspects of mental health, psychological symptoms, academic achievements, satisfaction and compliance with the course, and spirituality. The outcomes were mostly measured by self-reported questionnaires or essays, and in one study also interviews were included. There were no biological outcomes.

We defined the follow-up period as the timespan between the post-test assessment and follow-up assessment (given that there was also a baseline assessment before the course). Regarding the length of follow-up, 13 studies had no follow-up, two studies had a follow-up shorter than three months, five studies had a follow-up between three months and six months, three studies had a follow-up longer than six months (more specifically one year, one and half years and six years), and one study had a follow-up of unclear duration. A minority of the studies had several checkpoints during the follow-up period. The percentage of dropouts varied greatly due to the differences in the duration of follow-up. (Supplemental Digital Table S3).

### Characteristics of mindfulness interventions

The characteristics of the mindfulness interventions in all included studies are presented in Table 1.

**Table 1:** Description of the mindfulness interventions in included studies

Intervention	Description of the intervention and adaptations	Examples of typical meditations and retreat	Examples of practice at home	Obligatoriness and teacher of the course
8–10 weeks MBSR- or MBCT- based course (n = 6**)** (Danilewitz et al., 2016; Erogul et al., 2014; Malpass et al., 2019; Rosenzweig et al., 2003; Shapiro et al., 2019; van Dijk et al., 2017)	In general, the courses followed either MBSR or MBCT with some modifications of content, such as mindfulness in clinical situations, work life balance and student presentations about session themes. The sessions were shorter compared to original MBSR in some studies and the duration of weekly sessions ranged from 1 to 2 h	Body scan, loving kindness, sitting and walking meditation, raisin exercise, yoga/mindful movement, guided imagery/mountain meditation and awareness of breath. One course had a 5-h retreat day (Erogul et al., 2014)	In all interventions, students were encouraged to practice daily or 6 days/week at home. The length of mindfulness practice ranged from 20 to 30 min and some studies did not mention exact duration. Home practice included formal meditation and informal mindfulness exercises	None of the courses was mandatory part of the medical curriculum. Most of the courses were taught by trained mindfulness teachers. One study (Danilewitz et al., 2016) had a student peer leader who had received training in MBSR. One study did not mention the teacher (Rosenzweig et al., 2003)
4–7 weeks modified MBSR or MBCT course (n = 8) (Aherne et al., 2016; de Vibe et al., 2013; Garneau et al., 2013; Keng et al., 2015; Neto et al., 2020; Phang et al., 2015a, 2016; Shapiro et al., 1998)	The courses followed principally the content of MBSR or MBCT. The weekly sessions lasted mainly 2 h or longer (range 1–3 h). One course had sessions twice a week for four weeks (Garneau et al., 2013). Regarding content modifications, some of the courses had an emphasis on a specific theme e. g. practical side of mindfulness, informal practice or mindful communication	Body scan, raisin exercise, loving kindness, sitting and walking meditation, mindful breathing, nonjudgemental listening/communication, STOP, mindful movement/relaxation and empathy exercises. Two courses had a 6-h retreat day before the end of the course (de Vibe et al., 2013; Garneau et al., 2013)	In all the courses, students had home practice, mostly daily. The daily practice duration ranged from 3 to 30 min. The home exercises included both formal and informal practice. In addition, some courses had didactic homework	Most courses were not mandatory part of the medical curriculum. There were two mandatory courses (Aherne et al., 2016; Neto et al., 2020). Almost all courses were led by a trained mindfulness teacher, one study did not specify the teacher (Shapiro et al., 1998)
Non-specified mindfulness courses (n = 4**)** (Chung et al., 2018; Greeson et al., 2015; Kuhlmann et al., 2016; Moir et al., 2016)	Most courses were short in duration (4–5 weeks) and one course lasted for a semester (Moir et al., 2016). Session length ranged from 1 h to 1.5 h. The content of the sessions was mostly didactics and practice of mindfulness/mind–body medicine as well as group discussion. In some studies, the sessions were tailored for medical students in a specific environment (e. g. emergency medicine). One study did not describe mindfulness sessions in detail (Moir et al., 2016)	A variety of different exercises, such as short breathing and eating meditation, guided imagery, creative self-expression/drawing, loving kindness, body scan/awareness of the body and relaxation. No retreat days but one course had two social gatherings (Moir et al., 2016)	In all the interventions, students were encouraged to practice mindfulness at home (no exact time recommendations were mentioned nor type of exercise). In addition, some courses had home assignments	Most courses were not a mandatory part of the curriculum. One course was an integrated part of the emergency medicine rotations and therefore mandatory (Chung et al., 2018)
Mindfulness courses without face-to-face sessions (n = 5) (Danilewitz et al., 2018; Moore et al., 2020; Phang et al., 2015b; Warnecke et al., 2011; Yang et al., 2018)	The courses were carried out online (n = 2), via CD (n = 1), via DVD (n = 1) and via mobile phone application (n = 1). The course length varied mostly from 4 to 8 weeks. One online course (Danilewitz et al., 2018) had a flexible schedule and was possible to complete between 7 weeks and 4 months depending on the student. The courses were divided into modules or other types of weekly/daily sessions which included either didactics (e. g. about stress reduction, emotions and mindfulness in medical studies), mindfulness practice or both. One course was not described in detail (Warnecke et al., 2011)	A variety of different exercises, for example body scan, different types of breathing exercises, loving kindness, self-compassion, mountain meditation, mindful movement, mindful listening and eating and acknowledging thoughts	All the courses were completed remotely. The durations of different mindfulness exercises ranged from 5 to 20 min, though many studies did not specify the duration of mindfulness practice. Some courses had both informal and formal meditation	None of the courses were a mandatory part of the medical curriculum. The courses were completed independently and therefore had no teacher (however, there was a single meeting or briefing in some courses)
Compassion focused mindfulness course (n = 1) (Weingartner et al., 2019)	The course length varied between 5 and 8 weeks depending on the year. The sessions were 2 h long and included for example communication/listening practice, guided mindfulness meditation, compassion exercises and group discussion. (The content resembles the modified MBSR courses that have an emphasis on communication.)	For example: loving kindness meditation, listening and communicating exercises and compassion exercises	The recommendation was 15–30 min daily for home practice	Not mandatory part of the medical curriculum. The teacher was a trained instructor

MBSR = Mindfulness-based stress reduction, MBCT = Mindfulness-based cognitive therapy

Nineteen (79%) of the studies included an in-person/face-to-face mindfulness intervention and two (8%) studies were online interventions. Two (8%) studies used a CD or DVD mindfulness course and one (4%) study used a mindfulness mobile phone application.

Among the face-to-face interventions, six (32%) of the studies included the original or slightly modified 8-to 10-week MBSR or MBCT course, eight (42%) included a 4-to 7-week MBSR or MBCT-based course, one (5%) included a compassion-focused mindfulness course, three (16%) included 4- to 5-week non-specified mindfulness courses, and one (5%) included a one semester long non-specified mindfulness course. In most of the studies, the course was optional (n = 21); in a minority of the studies, the course was obligatory (n = 3) for the students. All the face-to-face interventions included home practice (n = 19).

Thirteen (68%) of the face-to-face interventions were led by a trained mindfulness teacher and three (16%) were led by peer students or a teacher with no official mindfulness training. The interventions without face-to-face sessions (n = 5) did not have a teacher. Three studies did not include information about the teacher.

### Mental health and psychological outcomes

The outcomes, assessment tools and summary of quantitative results are presented in Table 2.

**Table 2:** Analyzed outcomes, tools of assessment and summary of statistically significant results

Analyzed outcome	Tool of assessment (scale/measure)	Statistically significant results^a^
Stress (n = 15) (Danilewitz et al., 2016; de Vibe et al., 2013; Erogul et al., 2014; Garneau et al., 2013; Greeson et al., 2015; Keng et al., 2015; Kuhlmann et al., 2016; Moore et al., 2020; Neto et al., 2020; Phang et al., 2015a, 2015b, 2016; Shapiro et al., 2019; Warnecke et al., 2011; Yang et al., 2018)	The Perceived Stress Scale (PSS) Depression, Anxiety, and Stress Scale (DASS) Perceived Medical School Stress (PMSS) Trier Inventory for the Assessment of Chronic Stress (TICS)	Significant pre/post test results, decreases in stress, in 9/15 studies (in one or more subscales) Significant results in 3/8 of the follow-ups. Significant results in follow-ups lasting 1 month (Yang et al., 2018), 2 months (Warnecke et al., 2011) and 4 months (Moore et al., 2020). In two of these studies (Moore et al., 2020; Yang et al., 2018) significant changes at post-test assessment were not found, but a significant decrease in stress was found later at follow-up
Depression (n = 7) (Garneau et al., 2013; Keng et al., 2015; Moir et al., 2016; Neto et al., 2020; Shapiro et al., 1998, 2019; Warnecke et al., 2011)	The Depression, Anxiety and stress scale (DASS) Primary Health Questionnaire (PHQ-9) the Personal Health Questionnaire (PHQ-8) Hopkins Symptom Checklist 90 Revised (SCL-90-R) subscale 4 Beck Depression Inventory II (BDI-II)	Significant pre/post test results, decreases in depression, in 3/7 studies (in one or more subscales) Significant results in 0/1 of the follow-ups
Anxiety (n = 5) (Keng et al., 2015; Moir et al., 2016; Neto et al., 2020; Shapiro et al., 1998; Warnecke et al., 2011)	The Depression, Anxiety and stress scale (DASS)/(DASS-21) Generalized Anxiety Disorder questionnaire (GAD-7) The State-Trait Anxiety Inventory (Form Y)—STAI Form 1	Significant pre/post test results, decreases in anxiety, in 3/5 studies (in one or more subscales) The significant results persisted in 1/1 of the follow-ups (duration 8 weeks)
Psychological distress/general mental symptoms (n = 8) (de Vibe et al., 2013; Keng et al., 2015; Kuhlmann et al.,. 2016; Phang et al., 2015a, 2015b, 2016; Shapiro et al., 1998; van Dijk et al., 2017)	Depression, Anxiety, and Stress Scale (DASS) Brief Symptom Inventory (BSI) General Health Questionnaire (GHQ12) The Hopkins Symptom Checklist 90 Revised (SCL-90-R)	Significant pre/post test results, decreases in general mental symptoms, in 8/8 studies (in one or more subscales) The significant results persisted in 3/5 of the follow-ups. The significant results remained in follow-ups lasting 1 year (Kuhlmann et al.,. 2016), 1,5 years (van Dijk et al., 2017) and 6 years (Hanley et al., 2019)
Burnout (n = 3) (Danilewitz et al., 2018; de Vibe et al., 2013; Garneau et al., 2013)	the Maslach Burnout Inventory (MBI) Maslach Burnout Inventory-Human Services Survey (MBI-HSS)	Significant pre/post test results, decrease in burnout, in 1/3 studies (in one or more subscales) The significant results persisted in 0/1 of the follow-ups
Dysfunctional cognitions (n = 1) (van Dijk et al., 2017)	Irrational Beliefs Inventory (IBI)	1/1 had a significant result, decrease in dysfunctional cognitions, and significance persisted during 1,5 years of follow-up
Mindfulness/ mindfulness skills (n = 14) (Danilewitz et al., 2016, 2018; de Vibe et al., 2013; Garneau et al., 2013; Greeson et al., 2015; Keng et al., 2015; Neto et al., 2020; Phang et al., 2015a, 2015b, 2016; Shapiro et al., 2019; van Dijk et al., 2017; Weingartner et al., 2019; Yang et al., 2018)	The Five Facets of Mindfulness Questionnaire (FFMQ)/(FFMQ-SF) the Kentucky Inventory of Mindfulness Skills (KIMS) Mindful Awareness Attention Scale (MAAS) Cognitive and Affective Mindfulness Scale—Revised (CAMSR)	Significant pre/post test results, increases in mindfulness, in 11/14 studies (in one or more subscales) The significant results persisted in 2/6 of the studies with follow-ups. The significant results remained in follow-ups lasting 1,5 years (van Dijk et al., 2017) and 4 years (Solhaug et al., 2019)
Self-compassion (n = 5) (Danilewitz et al., 2016, 2018; Erogul et al., 2014; Garneau et al., 2013; Moore et al., 2020)	The Self-Compassion Scale (SCS)/(SCS-SF)	Significant pre/post test results, increases in self-compassion, in 5/5 studies (in one or more subscales) The significant results persisted in 2/2 of the follow-ups lasting 4 months (Moore et al., 2020) and 6 months (Erogul et al., 2014)
Empathy/Compassion (n = 6) (Danilewitz et al., 2016, 2018; Moore et al., 2020; Shapiro et al., 1998, 2019; van Dijk et al., 2017)	The Compassion Scale (CS) Jefferson Scale of Physician Empathy (JSE) the Jefferson Scale of Empathy-medical student version (JSE-S) Empathy Construct Rating Scale (ECRS)	Significant pre/post test results, increases in empathy, in 1/6 studies (in one or more subscales) There were significant results in 0/2 of the follow-ups
Altruism (n = 1) (Danilewitz et al., 2016)	the Adapted Altruism Scale	1/1 had a significant result, increase in altruism. No follow-up
Resilience/coping (n = 3) (Erogul et al., 2014; Kuhlmann et al., 2016; Moir et al., 2016)	Resilience Scale (RS), a 25-item resilience questionnaire and Brief COPE	Significant pre/post test results, increases in resilience, in 0/3 studies (in one or more subscales) There were significant results in 0/2 of the follow-ups
Subjective well-being/ Subjective happiness/ Psychological well-being/ general well-being/positive mental health (n = 6) (de Vibe et al., 2013; Garneau et al., 2013; Keng et al., 2015; Rosenzweig et al., 2003; van Dijk et al., 2017; Yang et al., 2018)	Subjective Well-Being scale (SWB) Profile of Mood States (POMS) Subjective Happiness Scale (SHS) General Well-Being Schedule (GWBS) Scales of Psychological Well-Being (SPWB) Mental Health Continuum-Short Form (MHC-SF)	Significant pre/post test results, increases in subjective well-being, in 5/6 studies (in one or more subscales) The significant results persisted in 3/3 of the follow-ups lasting 1 month, 1.5 years (van Dijk et al., 2017) and 6 years (de Vibe et al., 2013)
Self-efficacy (n = 2) (Phang et al., 2015a, 2015b)	General Self-efficacy Scale (GSE)	Significant pre/post test results in 2/2 studies (in one or more subscales) The significant results persisted in 1/2 of the follow-ups. The significant result remained in the follow-up that lasted 6 months (Phang et al., 2015a)
Academic skills/performance/motivation etc. (n = 2) (Moir et al., 2016; Shapiro et al., 2019)	Perceived Competence Scale the Motivated Strategies for Learning -Questionnaire a Likert-type educational outcomes survey	Significant pre/post test results in 0/2 studies (in one or more subscales). No follow-ups
Quality of life/Life satisfaction (n = 4) (Keng et al., 2015; Moir et al., 2016; Neto et al., 2020; van Dijk et al., 2017)	Linear Analogue Self-Assessment (LASA) World Health Organization Quality of Life (WHOQOL-Bref) Life Satisfaction Questionnaire (LiSat-9) Satisfaction With Life Scale (SWLS)	Significant pre/post test results, increases in life satisfaction, in 2/4 studies (in one or more subscales) The significant results persisted in 1/1 of the follow-ups lasting 1,5 years (van Dijk et al., 2017)
Spirituality (n = 1) (Shapiro et al., 1998)	The Index of Core Spiritual Experiences—INSPIRIT	1/1 had a significant result, increase in spirituality. No follow-up

^a^Results with *p*-value < 0.05 were considered statistically significant. i.e., significant

The studies used a variety of different self-reported outcome measures (Supplemental Digital Table S3).

The studies analyzed a large number of different outcomes. The most common outcomes were stress (n = 15), mindfulness (n = 14), mental distress (n = 8), depression (n = 7), empathy (n = 6), well-being (n = 6), self-compassion (n = 5) and anxiety (n = 5) (Table 2).

In more than 50% of the studies, the results were statistically significant regarding, for example, mindfulness (11 studies with significant results/14 studies that analyzed the outcome), stress (9/15), mental distress (8/8), well-being (5/6) and self-compassion (5/5). On the contrary, the results were statistically significant in less than 50% of the studies regarding outcomes such as depression (3/7) and empathy (1/6).

The results were similar in both controlled and uncontrolled studies. Regarding stress, results were statistically significant in 8 out of 12 controlled studies and in 3 out of 4 uncontrolled studies. In distress, the results were significant in 7/7 controlled and 1/1 uncontrolled studies. The corresponding numbers were 2/2 and 3/3 in self-compassion, 5/5 and 0/1 in well-being, and 6/9 and 5/5 in mindfulness. (Table 3).

**Table 3:** Effectiveness of different types of mindfulness interventions

Intervention	Effectiveness^a^	Outcomes with no statistically significant changes^a^
8–10 weeks MBSR- or MBCT- based course (n = 6) (Danilewitz et al., 2016; Erogul et al., 2014; Malpass et al., 2019; Rosenzweig et al., 2003; Shapiro et al., 2019; van Dijk et al., 2017)	Studies with this intervention type reported statistically significant changes in following outcomes (*studies with significant results/ studies that measured the outcome*): ● Stress *(2/4), all these studies had a control group* ● Mindfulness *(2/3), all these studies had a control group* ● Self-compassion *(2/2), both these studies had a control group* ● Distress *(1/1), this study had a control group* ● Quality of life *(1/1), this study had a control group* ● Well-being *(2/2), both these studies had a control group* ● Dysfunctional conditions *(1/1), this study had a control group* ● Altruism *(1/1), this study had a control group*	None of the studies with this intervention type reported significant changes in following outcomes *(number of studies that measured the outcome)*: ● Empathy *(3)* ● Resilience *(1)* ● Depression *(1)* ● Academic performance *(1)*
4–7 weeks modified MBSR or MBCT course (n = 8) (Aherne et al., 2016; de Vibe et al., 2013; Garneau et al., 2013; Keng et al., 2015; Neto et al., 2020; Phang et al., 2015a, 2016; Shapiro et al., 1998)	Studies with this intervention type reported significant changes in following outcomes (*studies with significant results/ studies that measured the outcome*): ● Stress *(4/6), in studies with control groups 3/4* ● Depression *(3/4), in studies with control groups 2/3* ● Mindfulness *(5/6), in studies with control groups 3/4* ● Anxiety *(2/3), all these studies had control groups* ● Self-compassion *(1/1), this study did not have a control group* ● Empathy *(1/1), this study had a control group* ● Burnout *(1/2), in studies with control groups 0/1* ● Distress *(5/5), in studies with control groups 4/4* ● Quality of life *(1/2), both these studies had control groups* ● Well-being *(2/3), in studies with control groups 2/2* ● Self-efficacy *(1/1), this study had a control group* ● Spirituality *(1/1), this study had a control group*	No outcomes without any significant changes
Non-specified mindfulness courses (n = 4) (Chung et al., 2018; Greeson et al., 2015; Kuhlmann et al., 2016; Moir et al., 2016)	Studies with this intervention type reported statistically significant changes in following outcomes (*studies with significant results/ studies that measured the outcome*): ● Stress *(1/2), in studies with control groups 0/1* ● Mindfulness *(1/1), this study did not have a control group* ● Distress *(1/1), this study had a control group*	None of the studies with this intervention type reported significant changes in following outcomes *(number of studies that measured the outcome)*: ● Depression *(1)* ● Anxiety *(1)* ● Quality of life *(1)* ● Resilience *(1)* ● Academic motivation *(1)* ● Coping *(1)*
Mindfulness courses without face-to-face sessions (n = 5) (Danilewitz et al., 2018; Moore et al., 2020; Phang et al., 2015b; Warnecke et al., 2011; Yang et al., 2018)	Studies with this intervention type reported significant changes in following outcomes (*studies with significant results/ studies that measured the outcome*): ● Stress *(4/4), in studies with control groups 3/3* ● Mindfulness *(2/3), in studies with control groups 1/2* ● Anxiety *(1/1), this study had a control group* ● Self-compassion *(2/2), neither of these studies had a control group* ● Distress *(1/1), this study had a control group* ● Well-being *(1/1), this study had a control group* ● Self-efficacy *(1/1), this study had a control group*	None of the studies with this intervention type reported significant changes in following outcomes *(number of studies that measured the outcome)*: ● Depression *(1)* ● Empathy/ Compassion *(2)* ● Burnout *(1)*
Compassion focused mindfulness course (n = 1) (Weingartner et al., 2019)	This study reported significant changes in mindfulness. *This study did not have a control group*	No outcomes without any significant changes

*MBSR* mindfulness-based stress reduction, *MBCT* mindfulness-based cognitive therapy

^a^Results with *p*-value < 0.05 were considered statistically significant. i.e., significant

It was possible to perform a meta-analysis for some of the outcomes: depression (number of studies = 4), anxiety (n = 4), stress (n = 9), distress (n = 5), mindfulness (n = 6), and well-being (including positive mental health) (n = 5). For other outcomes, there were fewer than 3 studies including a control group.

After the intervention, depression, and anxiety symptoms and well-being were not significantly different in the intervention group compared to controls (Supplemental Digital Figures). The intervention group had statistically significantly less symptoms of stress (Cohen’s d = −0.36, *p* < 0.001) and distress (Cohen’s d = −0.51, *p* = 0.007), and had higher mindfulness (Cohen’s d = 0.29, *p* = 0.004) compared to controls (Supplemental Digital Figures). Due to a low number of studies, it was not possible to analyze the effect of covariates.

There was low and statistically non-significant heterogeneity in outcomes of depression (*I*^2^ = 46.3%), anxiety (*I*^2^ = 0.1%), stress (*I*^2^ = 43.1%), and mindfulness (*I*^2^ = 37.4%). The heterogeneity was high for distress (*I*^2^ = 78.5%, *p* = 0.002) and well-being or positive mental health (*I*^2^ = 81.3%, *p* < 0.001). Based on Egger’s test, there was no indication of publication bias on any of the outcomes.

### Sustainability of effects: follow-up studies

There were five studies with months of follow-up (2015b; Erogul et al., 2014; Moore et al., 2020; Phang et al., 2015a; Warnecke et al., 2011). Four of these studies were RCTs (2015b; Erogul et al., 2014; Phang et al., 2015a; Warnecke et al., 2011) and one was a non-controlled study (Moore et al., 2020). In the studies with 2–6 months of follow-up, positive changes in stress (Moore et al., 2020; Warnecke et al., 2011) self-compassion (Erogul et al., 2014; Moore et al., 2020) and depression and anxiety (Warnecke et al., 2011) remained. In three studies, results regarding stress did not remain in 6-month follow-up (2015b; Erogul et al., 2014; Phang et al., 2015a).

Two studies with years of follow-up were found (de Vibe et al., 2018; van Dijk et al., 2017). In a randomized controlled trial, 144 Norwegian medical (approximately 60% of the sample) and psychology students received a 7-week mindfulness course and a control group of 144 continued with their study curriculum (de Vibe et al., 2018). At the six-year follow-up, the students receiving mindfulness training reported greater increase in well-being, dispositional mindfulness, and problem-focused coping and greater decrease in avoidance-focused coping when compared to the controls (de Vibe et al., 2018). In a cluster-randomized controlled trial comparing clerkships with additional MBSR (n = 83) to clerkships as usual (CAU, n = 84) in medical students, the MBSR group reported a decrease in psychological distress and an increase in positive mental health, life satisfaction, and mindfulness skills compared with CAU during the 20-month follow-up (van Dijk et al., 2017).

### Satisfaction and compliance

Program satisfaction was assessed in nine studies (2015b; Aherne et al., 2016; Chung et al., 2018; Danilewitz et al., 2016, 2018; Garneau et al., 2013; Greeson et al., 2015; Phang et al., 2015a; Rosenzweig et al., 2003). Several different assessment tools were used, such as closed-ended questions (yes/no), a satisfaction score, and Likert-type scales (agree/disagree). All of these studies reported that most of the students were satisfied with the course or found the course beneficial/effective and would recommend it to others. In addition, optional course students were (statistically significantly) more satisfied with the course than mandatory course students (Aherne et al., 2016).

Compliance was assessed in 17 studies (2015b; Aherne et al., 2016; Danilewitz et al, 2016, 2018; de Vibe et al., 2013; Erogul et al., 2014; Greeson et al., 2015; Kuhlmann et al., 2016; Moir et al., 2016; Moore et al., 2020; Phang et al., 2015a, 2016; Shapiro et al., 1998, 2019; van Dijk et al., 2017; Warnecke et al., 2011; Yang et al., 2018). In these studies, full attendance to the course sessions (e.g. 7/7 sessions) ranged from 49 to 77%. The average percentage of attended sessions varied between 49 and 93%. Mean of days of mindfulness practice at home per week during the intervention ranged from 1.5 to 3.3 days. The time spent on one meditation session was on average less than 15 min.

Some studies reported that the duration of mindfulness practice at home (Moore et al., 2020) or the number of students who did meditation at home (van Dijk et al., 2017) declined already during the course. At follow-up, a decline was seen both in the number of students doing meditation (Moore et al., 2020; van Dijk et al., 2017; Yang et al., 2018) and the amount of mindfulness practice (2015b; Phang et al., 2015a).

### Effectiveness of different types of mindfulness interventions

The effectiveness of different types of mindfulness courses is described in Table 3. The effectiveness of the 4- to 7-week courses did not differ greatly from that of the 8- to 10-week courses. In addition, some types of courses without face-to-face sessions can be effective as well. There were significant effects in both uncontrolled and controlled study designs regarding several outcomes (Table 3).

### Qualitative results

There were five mixed methods studies with qualitative data (Aherne et al., 2016; Greeson et al., 2015; Moore et al., 2020; Weingartner et al., 2019; Yang et al., 2018). They used several different ways of data collection, such as open-ended questions and essays. In addition, one study included only qualitative data (Malpass et al., 2019).

A full description of the results of thematic synthesis is presented in Supplement Digital Content. In summary, six broad themes were identified: Increased awareness and mindfulness; Increased well-being, resilience and balance; Kindness and compassion towards others and self; Work or studies-related benefits; Factors affecting feasibility and satisfaction; and Difficulties and adverse effects (Supplement Digital Content, *Qualitative results*).

After the mindfulness intervention, increased awareness was constantly reported, towards oneself (e.g. Greeson et al., 2015), others (e.g.Garneau et al., 2013), own thoughts (Malpass et al., 2019; Moore et al., 2020), emotions, body and behavior (e.g. Moore et al., 2020). Greater awareness led to earlier recognition of stress and better possibilities to manage stress and regulate own behavior (Malpass et al., 2019; Moore et al., 2020). Increased awareness of negative thoughts, in a non-judgmental way, was important for building a new relationship with own thoughts and feelings (Moore et al., 2020). There were also some experiences of enhanced awareness towards own body (Garneau et al., 2013).

Participants of mindfulness interventions reported many beneficial changes in well-being and symptoms of mental distress, for example, less judgment (Moore et al., 2020), decreased comparing to others (Malpass et al., 2019), increased acceptance (Garneau et al., 2013; Malpass et al., 2019; Moore et al., 2020), concentration (Malpass et al., 2019), patience (Weingartner et al., 2019), and happiness (Malpass et al., 2019; Weingartner et al., 2019). They felt a better sense of balance (Greeson et al., 2015) and control, resilience and flexibility, and better ways of coping (Malpass et al., 2019) and managing stress (Greeson et al., 2015).

They learned the means and appreciation of self-care (Greeson et al., 2015). Enhanced compassion in general, and especially towards patients was reported (Moore et al., 2020; Weingartner et al., 2019). Learning acceptance was an important factor relating to self-compassion (Malpass et al., 2019; Moore et al., 2020).

Increased concentration and efficiency (Malpass et al., 2019) were experienced. Increased awareness and via this, change in working habits were related to better efficiency (Malpass et al., 2019). Mindfulness increased compassion toward patients, and also enhanced the sense of connection with others (Garneau et al., 2013; Weingartner et al., 2019). Participants reported how they used the learned skills and techniques when working in the hospital with deadlines (Moore et al., 2020) and towards the patients (Weingartner et al., 2019).

Participants were generally satisfied with mindfulness interventions. The right timing of the course was important (Aherne et al., 2016). The content, amount, and timing of meditations during the course affects the feasibility (Aherne et al., 2016; Moore et al., 2020). In one study, comparing optional and mandatory mindfulness courses, the mandatory course was not seen as beneficial (Aherne et al., 2016). Kind and genuine teachers had a significant role (Aherne et al., 2016; Weingartner et al., 2019). The significance of peer group was evident, offering a safe, accepting, and warm place to bond and develop (Malpass et al., 2019)*.*

Also, some difficulties were reported. Noticing self-judgmental feelings was difficult and may cause anxiety (Malpass et al., 2019). Some students found it difficult to sit still (Aherne et al., 2016), and it may be difficult to relax and sit still especially when stressed (Moore et al., 2020).

Adverse effects were not asked specifically, but some of the participants reported challenges and increased stress (Malpass et al., 2019). Especially mandatory mindfulness courses may increase stress (Aherne et al., 2016).

### Quality of the included studies

The quality rating of included studies is presented in detail in Supplemental Digital Table S4a-b. In general, the quality of studies was variable and none of the studies was excluded due to poor quality. Among studies with quantitative data, MERSQI scores ranged from 6 to 14 (the highest possible score in MERSQI is 18), and the mean score was 12. The vast majority of the studies were single-center studies. In 3/23 of the studies, the response rate was very low (under 50%). Most of the studies used validated outcome assessment scales. All the studies used only self-reported outcome data from the participants. None of the studies measured objectively the effect of a mindfulness intervention on e.g. academic performance or performance in clinical work. In most of the studies, statistical analyses were considered appropriate. Among studies with qualitative data, the quality was in general adequate, though variable. A philosophical perspective was rarely presented and the researchers were not located culturally or theoretically. Also, the influence of the researcher on the research, and vice-versa was mostly unclear.

## Discussion

### Main results

The number of studies on mindfulness interventions in medical students has increased drastically during the last ten years, and the samples are from all around the world. Of all included studies, 54% were published after 2015. Over half of the studies were RCTs. The sample sizes of the studies were relatively small. Most of the studies analyzed only pre-post differences whereas studies with long follow-up (several months or years) were few. In over half of the studies, the intervention was from 4 to 10 weeks long, either an original MBSR or MBCT-based course or a modification of this. In the vast majority of the studies, mindfulness courses were optional for the students.

The studies analyzed several outcomes, such as stress and mindfulness, mental distress, depression, empathy, well-being, self-compassion, and anxiety, and most of the outcomes were based on self-reported questionnaires. In individual studies, there were statistically significant beneficial changes in mindfulness, stress, mental distress, well-being, and self-compassion. There were significant results in controlled and uncontrolled studies.

Based on a meta-analysis, after the intervention, depression and anxiety symptoms and well-being did not differ in the intervention group compared to controls. The intervention group had statistically significantly less symptoms of stress and distress and had higher mindfulness compared to the controls. The size of the effect on stress and distress was small and to mindfulness moderate. Due to a low number of controlled studies, it was not possible to perform a meta-analysis for all the outcomes, and it was not possible to analyze the effect of covariates.

The effectiveness of the 4- to 7-week modified MBSR courses did not differ greatly from that of the 8- to 10-week MBSR-based courses. Courses without face-to-face sessions were effective as well. Positive changes in self-compassion and stress remained in the months of follow-up. In two studies with more than 1.5 years and 6 years of follow-up, the results regarding well-being and mental distress remained unchanged.

### Comparison to earlier studies and methodological and theoretical discussion

McConville et al. (2017) performed a meta-analysis on the effectiveness of mindfulness interventions in controlled trials in health profession students, including 10 studies with medical students. Their results indicated that mindfulness-based interventions decrease stress, anxiety, and depression and improve mindfulness and mood in health profession students. Their results were not presented separately for medical students and there were thus no overlapping studies with our study.

A systematic review by Daya and Hearn (2018) included 12 studies on the effects of mindfulness on medical students’ stress, depression, fatigue, and burnout. They found that during the mindfulness interventions stress and depression decreased statistically significantly. Very variable mindfulness interventions were included, also well-being interventions with mindfulness as only part of the intervention. Our study included 7 overlapping studies with the review of Daya and Hearn (2018). However, our study is novel due to including more studies and analyzing several outcomes, being able to perform a meta-analysis, and also due to including an analysis of qualitative data. Still, the conclusion of their and our study are similar. It could be beneficial to incorporate mindfulness programs in medical schools since it would offer another way to enhance medical students’ psychological well-being.

Also based on our study, mindfulness interventions have a significant effect on reducing stress, also in meta-analysis. Although our review included some original studies showing beneficial effects on anxiety and depression, these studies were relatively few compared to other outcomes and there was no significant effect in the meta-analysis. Our review extends the results of previous reviews on the effects of mindfulness to several other outcomes. We found that in addition to decreasing stress, mindfulness interventions can be beneficial for mindfulness and mental distress.

Our thematic synthesis adduced potential explanations for the quantitative results. There are possibly many explanations for decreased stress and distress. Increased awareness may lead to earlier recognition of stress and better possibilities to manage stress and regulate own behavior (Malpass et al., 2019; Moore et al., 2020). Increased awareness of negative thoughts and acceptance was important for building a new relationship with own thoughts and feelings (Moore et al., 2020). Mindfulness may lead to less judgment, less comparison to others, and increased acceptance (Garneau et al., 2013; Malpass et al., 2019; Moore et al., 2020). These may lead to a greater sense of balance (Greeson et al., 2015) and control, resilience, and flexibility (Malpass et al., 2019).

The number of studies analyzing self-compassion was too few for performing a meta-analysis, but in individual controlled and uncontrolled studies, mindfulness increased self-compassion (Danilewitz et al., 2016, 2018; Erogul et al., 2014; Garneau et al., 2013; Moore et al., 2020). This may have positive effects on a person’s well-being later in life. For example, in a sample of more than 2,000 pediatric residents, higher self-compassion predicted a lower risk of stress at follow-up (Kemper et al., 2020). Based on qualitative synthesis, in mindfulness courses, students learned means and appreciation of self-care (Greeson et al., 2015), and acceptance was an important factor relating to self-compassion (Malpass et al., 2019; Moore et al., 2020).

In all but one study, the analyzed outcomes were based on self-reported questionnaires or essays, and no physiological measures were analyzed. In one study, also interviews were included (Malpass et al., 2019). Some of the effects of mindfulness interventions may be difficult to capture with outcome scales. As in psychotherapy research (Blatt et al., 2000), the measures used in the studies may not assess qualitative and developing phenomena occurring during or after the mindfulness course. Outcomes of this kind are very difficult to capture by structured scales. In later studies, it would be interesting to see the effects of mindfulness on well-being based on blinded interviews. In addition, it would be interesting and important to analyze outcomes such as educational functioning, academic achievements, and work performance and satisfaction.

Based on thematic synthesis, there were many beneficial changes relating to work and studies. Students reported increased awareness, change in working habits, and better efficiency (Malpass et al., 2019). They used the learned skills and techniques when working in the hospital (Moore et al., 2020). Mindfulness also increased compassion toward patients (Garneau et al., 2013; Weingartner et al., 2019). None of the included studies used objective measures of academic or work performance, and this should be considered in future studies on the effects of mindfulness.

There are still few studies analyzing the long-term effects of mindfulness among medical students. Five studies with 2 to 6 months of follow-up (2015b; Erogul et al., 2014; Moore et al., 2020; Phang et al., 2015a; Warnecke et al., 2011) and two studies with approximately 1.5 to 6 years of follow-up were found (de Vibe et al., 2018; van Dijk et al., 2017). Previous systematic reviews did not report results on the persistence of changes over months or years of follow-up. Based on our systematic review, in some studies, positive changes in stress (Moore et al., 2020; Warnecke et al., 2011), self-compassion (Erogul et al., 2014; Moore et al., 2020) and depression and anxiety (Warnecke et al., 2011) persisted in the follow-up months. However, in two studies the results regarding stress did not remain in the 6-month follow-up (Erogul et al., 2014; Phang et al., 2015b). At the six-year follow-up, the students receiving mindfulness training reported greater increased well-being, dispositional mindfulness and problem-focused coping and a greater decrease in avoidance-focused coping when compared to controls (de Vibe et al., 2018). In one study with a 20-month follow-up, students in the mindfulness group showed a decrease in psychological distress and increase in positive mental health, life satisfaction, and mindfulness skills compared with controls (van Dijk et al., 2017).

Only one study analyzing a mindfulness course focusing on compassion was found (Weingartner et al., 2019). This small study without a control group showed a significant increase in mindfulness skills after the course. In the future, analyzing the effects of compassion-focused mindfulness interventions on a variety of outcomes would be useful.

### Practical implications

Our systematic review highlights some practical implications. All structured mindfulness courses seem to be beneficial regarding medical students’ mental health, mindfulness skills, stress symptoms, well-being and self-compassion. The length of the course (from 4 to 10 weeks) did not seem to affect the results, and also courses that were not delivered face to face had beneficial effects. In contrast, the mindfulness courses with less structured content or which were not described in detail had more often non-significant results.

The participants were generally satisfied with mindfulness interventions. Some factors affecting feasibility and satisfaction were found. In one study comparing optional and mandatory mindfulness courses, students in the optional course were more satisfied with the course and students in the mandatory course gave critical feedback (Aherne et al., 2016). It is likely that specifically optional mindfulness courses are effective in enhancing the well-being of students.

The right timing of the course is important (Aherne et al., 2016). On the other hand, it may be that there never may be a right time for self-care within medical training. If this is true, it indicates a systematic problem within medical education (Aherne et al., 2016) that needs to be discussed and handled.

The instructor seems to be important for the success of the course. The students appreciated kind and genuine teachers, who were also role models (Aherne et al., 2016; Weingartner et al., 2019). Two studies had peer instructors in their mindfulness courses (Danilewitz et al., 2016; Moir et al., 2016). It would be important to study further the role of the instructor on the effectiveness of the course. If courses with peer instructors were shown to have similar outcomes as courses with a trained mindfulness teacher, it would enhance the possibility to offer effective mindfulness interventions due to practical and financial limitations.

Learning and practicing mindfulness with peer group is beneficial. A group offers a safe, accepting, and warm place to bond and develop (Malpass et al. (2019), and discuss the challenges of the developing physician.

Although none of the included studies assessed the harms or side effects of mindfulness interventions systematically, some harmful effects and experiences were reported. For example, difficulties in dealing with (judgmental) thoughts, irritation, getting disturbed by others in the group, bad feelings due to difficulties in relaxing or difficulties in experiencing own body, difficulties in sitting still or relaxing. Some of the participants reported challenges and increased stress (Malpass et al., 2019). Especially mandatory mindfulness course may increase stress (Aherne et al., 2016).

Based on recent studies with other than medical student samples, 83–87% of persons experience momentary harms of mindfulness, most commonly anxiety, and 6–25% may experience lasting harmful effects (Aizik-Reebs et al., 2021). The lasting effects may relate to increased awareness of the internal states (Aizik-Reebs et al., 2021; Britton et al., 2021), without the proper skill to manage and accept the experiences. These results highlight the importance of trained instructors, highlighting the learning of acceptance and management of difficult thoughts, feelings, and experiences. Also, including aspects of compassion and self-compassion are important. Offering students the possibility to have a personal discussion with the instructor, and the development of sustainability of mindfulness practice after the course would be important as well.

### Strengths and limitations

Our study has many strengths. We did not exclude any outcomes and therefore we were able to collect and analyze a large amount of information on the effects of mindfulness interventions. Both quantitative and qualitative studies were included. In addition, we wanted to include all types of interventions that had mindfulness as their main component to get a good overview of the research on mindfulness on medical students so far. We collected and presented detailed information on mindfulness interventions in the included studies. The results were also inspected by the characteristics of the mindfulness interventions (i.e., length of the course). Presenting the characteristics of the mindfulness courses and their results may be beneficial for developing future studies and also for developing mindfulness courses in medical schools. Our literature search from databases was extensive, covering five databases. The literature search was approved by an informatician.

There were also some limitations. Due to practical issues, we were only able to include studies in English, which means that some important findings may have been left out. In addition, we wanted to concentrate on mindfulness courses so interventions with only one session were excluded. The studies included in our systematic review varied greatly in terms of methods and outcome measures, which created challenges in collating the data. The quality of the included studies and their reporting varies. However, in general, the quality of the included studies was adequate. For example, the mean MERSQI score (12) was comparable to that of other studies on medical education (Cook et al., 2015). Many of the studies had small sample size. Some of the studies did not have a control group. Some of the studies did not report detailed background information of the samples, and it seems that the sample in some studies was not originally planned for scientific research. Due to the relatively low number of studies, meta-analysis was possible to perform only for some of the outcomes, and covariates could not be analyzed. Though there was no indication of publication bias, the low number of studies decreases the reliability of this result.

## Conclusions

The number of studies on mindfulness interventions for medical students has increased drastically during the last ten years. Mindfulness-based interventions seem to have beneficial effects on medical students’ stress, mental distress, mindfulness, and potentially also to well-being and self-compassion. There is some tentative indication of the persistence of effects in long-term follow-up. The effects of mindfulness on objectively measured academic and work performance have not been studied yet. In addition, the studies so far have not included a systematic assessment of the potential harms and adverse effects of mindfulness. In the future, there is a need for studies with large samples, rigorous methods, years of follow-up, and variable outcome measures to shed more light on the effects of mindfulness.

### Supplementary Information

Below is the link to the electronic supplementary material.

Supplementary file1 (DOCX 249 kb)
