# Supplementary material for: The effects of mindfulness-based interventions in medical students: a systematic review
Source: Adv Health Sci Educ Theory Pract. 2023 May 25;29(1):245–71. doi: 10.1007/s10459-023-10231-0 (PMC10927869; doi:10.1007/s10459-023-10231-0)
Supplement: Supplementary file 1 — Supplementary file1 (DOCX 249 kb) [file 10459_2023_10231_MOESM1_ESM.docx]

**Supplemental Digital Content Table 1a.** Prisma Checklist.

| **Section and Topic** | **Item #** | **Checklist item** | **Location where item is reported** |
| --- | --- | --- | --- |
| **TITLE** | | |  |
| Title | 1 | Identify the report as a systematic review. | Title page |
| **ABSTRACT** | | |  |
| Abstract | 2 | See the PRISMA 2020 for Abstracts checklist. | X |
| **INTRODUCTION** | | |  |
| Rationale | 3 | Describe the rationale for the review in the context of existing knowledge. | Pages 1-2 |
| Objectives | 4 | Provide an explicit statement of the objective(s) or question(s) the review addresses. | Page 3 |
| **METHODS** | | |  |
| Eligibility criteria | 5 | Specify the inclusion and exclusion criteria for the review and how studies were grouped for the syntheses. | Page 3 |
| Information sources | 6 | Specify all databases, registers, websites, organisations, reference lists and other sources searched or consulted to identify studies. Specify the date when each source was last searched or consulted. | Page 3 |
| Search strategy | 7 | Present the full search strategies for all databases, registers and websites, including any filters and limits used. | SDC table 2 |
| Selection process | 8 | Specify the methods used to decide whether a study met the inclusion criteria of the review, including how many reviewers screened each record and each report retrieved, whether they worked independently, and if applicable, details of automation tools used in the process. | Page 4 |
| Data collection process | 9 | Specify the methods used to collect data from reports, including how many reviewers collected data from each report, whether they worked independently, any processes for obtaining or confirming data from study investigators, and if applicable, details of automation tools used in the process. | Page 4 |
| Data items | 10a | List and define all outcomes for which data were sought. Specify whether all results that were compatible with each outcome domain in each study were sought (e.g. for all measures, time points, analyses), and if not, the methods used to decide which results to collect. | Page 4 |
|  | 10b | List and define all other variables for which data were sought (e.g. participant and intervention characteristics, funding sources). Describe any assumptions made about any missing or unclear information. | Page 4 |
| Study risk of bias assessment | 11 | Specify the methods used to assess risk of bias in the included studies, including details of the tool(s) used, how many reviewers assessed each study and whether they worked independently, and if applicable, details of automation tools used in the process. | SDC tables 4 |
| Effect measures | 12 | Specify for each outcome the effect measure(s) (e.g. risk ratio, mean difference) used in the synthesis or presentation of results. | Page 5 |
| Synthesis methods | 13a | Describe the processes used to decide which studies were eligible for each synthesis (e.g. tabulating the study intervention characteristics and comparing against the planned groups for each synthesis (item #5)). | Page 5 |
|  | 13b | Describe any methods required to prepare the data for presentation or synthesis, such as handling of missing summary statistics, or data conversions. | Not applicable |
|  | 13c | Describe any methods used to tabulate or visually display results of individual studies and syntheses. | Page 5 |
|  | 13d | Describe any methods used to synthesize results and provide a rationale for the choice(s). If meta-analysis was performed, describe the model(s), method(s) to identify the presence and extent of statistical heterogeneity, and software package(s) used. | Page 5 |
|  | 13e | Describe any methods used to explore possible causes of heterogeneity among study results (e.g. subgroup analysis, meta-regression). | Not applicable |
|  | 13f | Describe any sensitivity analyses conducted to assess robustness of the synthesized results. | Not applicable |
| Reporting bias assessment | 14 | Describe any methods used to assess risk of bias due to missing results in a synthesis (arising from reporting biases). | SDC Table 3 |
| Certainty assessment | 15 | Describe any methods used to assess certainty (or confidence) in the body of evidence for an outcome. | Not applicable |
| **RESULTS** | | |  |
| Study selection | 16a | Describe the results of the search and selection process, from the number of records identified in the search to the number of studies included in the review, ideally using a flow diagram. | Figure 1 |
|  | 16b | Cite studies that might appear to meet the inclusion criteria, but which were excluded, and explain why they were excluded. | Not presented |
| Study characteristics | 17 | Cite each included study and present its characteristics. | Pages 6-7, SDC Table 3 |
| Risk of bias in studies | 18 | Present assessments of risk of bias for each included study. | SDC Tables 4 |
| Results of individual studies | 19 | For all outcomes, present, for each study: (a) summary statistics for each group (where appropriate) and (b) an effect estimate and its precision (e.g. confidence/credible interval), ideally using structured tables or plots. | Tables 2 and 3,  SDC Figures |
| Results of syntheses | 20a | For each synthesis, briefly summarise the characteristics and risk of bias among contributing studies. | Not applicable |
|  | 20b | Present results of all statistical syntheses conducted. If meta-analysis was done, present for each the summary estimate and its precision (e.g. confidence/credible interval) and measures of statistical heterogeneity. If comparing groups, describe the direction of the effect. | Tables 2 and 3,  SDC Figures |
|  | 20c | Present results of all investigations of possible causes of heterogeneity among study results. | Not applicable |
|  | 20d | Present results of all sensitivity analyses conducted to assess the robustness of the synthesized results. | Not applicable |
| Reporting biases | 21 | Present assessments of risk of bias due to missing results (arising from reporting biases) for each synthesis assessed. | Not applicable |
| Certainty of evidence | 22 | Present assessments of certainty (or confidence) in the body of evidence for each outcome assessed. | Not applicable |
| **DISCUSSION** | | |  |
| Discussion | 23a | Provide a general interpretation of the results in the context of other evidence. | Pages 20-23 |
|  | 23b | Discuss any limitations of the evidence included in the review. | Pages 20-23 |
|  | 23c | Discuss any limitations of the review processes used. | Page 23 |
|  | 23d | Discuss implications of the results for practice, policy, and future research. | Pages 22-23 |
| **OTHER INFORMATION** | | |  |
| Registration and protocol | 24a | Provide registration information for the review, including register name and registration number, or state that the review was not registered. | Not applicable |
|  | 24b | Indicate where the review protocol can be accessed, or state that a protocol was not prepared. | Not prepared |
|  | 24c | Describe and explain any amendments to information provided at registration or in the protocol. | Not applicable |
| Support | 25 | Describe sources of financial or non-financial support for the review, and the role of the funders or sponsors in the review. | No funders |
| Competing interests | 26 | Declare any competing interests of review authors. | No conflict of interest |
| Availability of data, code and other materials | 27 | Report which of the following are publicly available and where they can be found: template data collection forms; data extracted from included studies; data used for all analyses; analytic code; any other materials used in the review. | Not applicable |

**Supplemental Digital Content Table 1b.** Prisma Checklist for Abstracts.

| **Section and Topic** | **Item #** | **Checklist item** | **Reported (Yes/No)** |
| --- | --- | --- | --- |
| **TITLE** | | |  |
| Title | 1 | Identify the report as a systematic review. | Yes |
| **BACKGROUND** | | |  |
| Objectives | 2 | Provide an explicit statement of the main objective(s) or question(s) the review addresses. | Yes |
| **METHODS** | | |  |
| Eligibility criteria | 3 | Specify the inclusion and exclusion criteria for the review. | Yes |
| Information sources | 4 | Specify the information sources (e.g. databases, registers) used to identify studies and the date when each was last searched. | No |
| Risk of bias | 5 | Specify the methods used to assess risk of bias in the included studies. | No |
| Synthesis of results | 6 | Specify the methods used to present and synthesise results. | No |
| **RESULTS** | | |  |
| Included studies | 7 | Give the total number of included studies and participants and summarise relevant characteristics of studies. | Yes |
| Synthesis of results | 8 | Present results for main outcomes, preferably indicating the number of included studies and participants for each. If meta-analysis was done, report the summary estimate and confidence/credible interval. If comparing groups, indicate the direction of the effect (i.e. which group is favoured). | Yes |
| **DISCUSSION** | | |  |
| Limitations of evidence | 9 | Provide a brief summary of the limitations of the evidence included in the review (e.g. study risk of bias, inconsistency and imprecision). | No |
| Interpretation | 10 | Provide a general interpretation of the results and important implications. | Yes |
| **OTHER** | | |  |
| Funding | 11 | Specify the primary source of funding for the review. | Yes |
| Registration | 12 | Provide the register name and registration number. | No |

**Supplemental digital content Table 1c.** ENTREQ statement items

| **No Item** | **Guide and description** | **Check** |
| --- | --- | --- |
|  |  |  |
| 1  Aim | State the research question the synthesis addresses. | X |
| 2  Synthesis methodology | Identify the synthesis methodology or theoretical framework which underpins the synthesis, and describe the rationale for choice of methodology (e.g. meta-ethnography, thematic synthesis, critical interpretive synthesis, grounded theory synthesis, realist synthesis, meta-aggregation, meta-study, framework synthesis). | X |
|  |  |  |
|  |  |  |
| 3  Approach to searching | Indicate whether the search was pre-planned (comprehensive search strategies to seek all available studies) or iterative (to seek all available concepts until they theoretical saturation is achieved). | X |
|  |  |  |
| 4  Inclusion criteria | Specify the inclusion/exclusion criteria (e.g. in terms of population, language, year limits, type of publication, study type). | X |
|  |  |  |
| 5  Data sources | Describe the information sources used (e.g. electronic databases (MEDLINE, EMBASE, CINAHL, psycINFO, Econlit), grey literature databases (digital thesis, policy reports), relevant organisational websites, experts, information specialists, generic web searches (Google Scholar) hand searching, reference lists) and when the searches conducted; provide the rationale for using the data sources. | X |
|  |  |  |
| 6  Electronic Search strategy | Describe the literature search (e.g. provide electronic search strategies with population terms, clinical or health topic terms, experiential or social phenomena related terms, filters for qualitative research, and search limits). | X |
|  |  |  |
| 7  Study screening methods | Describe the process of study screening and sifting (e.g. title, abstract and full text review, number of independent reviewers who screened studies). | X |
|  |  |  |
| 8  Study characteristics | Present the characteristics of the included studies (e.g. year of publication, country, population, number of participants, data collection, methodology, analysis, research questions). | X |
|  |  |  |
| 9  Study selection results | Identify the number of studies screened and provide reasons for study exclusion (e,g, for comprehensive searching, provide numbers of studies screened and reasons for exclusion indicated in a figure/flowchart; for iterative searching describe reasons for study exclusion and inclusion based on modifications t the research question and/or contribution to theory development). | X |
|  |  |  |
| 10  Rationale for appraisal | Describe the rationale and approach used to appraise the included studies or selected findings (e.g. assessment of conduct (validity and robustness), assessment of reporting (transparency), assessment of content and utility of the findings). | X |
|  |  |  |
| 11  Appraisal items | State the tools, frameworks and criteria used to appraise the studies or selected findings (e.g. Existing tools: CASP, QARI, COREQ, Mays and Pope [25]; reviewer developed tools; describe the domains assessed: research team, study design, data analysis and interpretations, reporting). | X |
|  |  |  |
| 12  Appraisal process | Indicate whether the appraisal was conducted independently by more than one reviewer and if consensus was required. | X |
|  |  |  |
| 13  Appraisal results | Present results of the quality assessment and indicate which articles, if any, were weighted/excluded based on the assessment and give the rationale. | X |
|  |  |  |
| 14  Data extraction | Indicate which sections of the primary studies were analysed and how were the data extracted from the primary studies? (e.g. all text under the headings “results /conclusions” were extracted electronically and entered into a computer software). | X |
|  |  |  |
| 15  Software | State the computer software used, if any. | X |
|  |  |  |
| 16  Number of reviewers | Identify who was involved in coding and analysis. | X |
|  |  |  |
| 17  Coding | Describe the process for coding of data (e.g. line by line coding to search for concepts). | X |
|  |  |  |
| 18  Study comparison | Describe how were comparisons made within and across studies (e.g. subsequent studies were coded into pre-existing concepts, and new concepts were created when deemed necessary). | X |
|  |  |  |
| 19  Derivation of themes | Explain whether the process of deriving the themes or constructs was inductive or deductive. | X |
|  |  |  |
| 20  Quotations | Provide quotations from the primary studies to illustrate themes/constructs, and identify whether the quotations were participant quotations or the author’s interpretation. | X |
|  |  |  |
| 21  Synthesis output | Present rich, compelling and useful results that go beyond a summary of the primary studies (e.g. new interpretation, models of evidence, conceptual models, analytical framework, development of a new theory or construct). | X |

**Supplemental digital content Table 2.** Full search strategy.

**PubMed search strategy, June 2020**

(("Mindfulness"[Mesh]) OR (mindfulness [Text Word])) AND (("Students, Medical"[Mesh]) OR (medic*[Text Word] AND student*[Text Word]))

Text words= Includes all words and numbers in the title, abstract, other abstract, MeSH terms, MeSH Subheadings, Publication Types, Substance Names, Personal Name as Subject, Corporate Author, Secondary Source, Comment/Correction Notes, and Other Terms (see Other Term [OT] above) typically non-MeSH subject terms (keywords), including NASA Space Flight Mission, assigned by an organization other than NLM.

**Scopus search strategy, June 2020**

TITLE-ABS-KEY (mindfulness) AND TITLE-ABS-KEY (medic* AND student*)

TITLE-ABS-KEY= A combined field that searches abstracts, keywords, and document titles.

**CINAHL search strategy, June 2020**

( (MH "Mindfulness") OR mindfulness ) AND ( (MH "Students, Medical") OR ( medic* AND student* ) )

Expanders: Apply equivalent subjects. Limiters: Scholarly (Peer reviewed) Journals.

When performing a search in the CINAHL databases, the default fields for unqualified searches are: Title, Abstract, Subject headings, PubMed ID (PMID), Digital Object Identifier (DOI) and Author

**PsycArticles search strategy, June 2020**

mindfulness AND medic* AND student*

Expanders: Apply equivalent subjects. Limiters: Scholarly (Peer reviewed) Journals.

The default fields for unqualified searches consist of the following: Title, Translated Title, Abstract, Keyword, Subjects, Table of Contents, and Author.

**Web Of Science search strategy, June 2020**

TS=(mindfulness) AND TS=(medic* AND student*)

Document types: Article.

TS=Topic= searches the following fields within a record: Title, Abstract, Author Keywords and Keywords Plus.

**Education Collection (ProQuest) search strategy, June 2020**

noft(mindfulness) AND noft(medic* AND student*)

Limit to: peer reviewed.

Noft= Anywhere except full text

**Supplemental Digital Content Table 3.** Description of included studies.

| **Authors and country**  **Study design** | **Sample size**  **Drop-out/attrition**  **Number of women**  **Age of the sample** | **Inclusion-/exclusion criteria**  **The phase of medical studies** | **Measured outcomes and their scales** | **Mindfulness-course (programme)**  **Duration**  **Number and duration of meetings Obligatoriness**  **Course leader**  **Duration of the intervention and the study** |
| --- | --- | --- | --- | --- |
| Aherne et al. 2016  Ireland  A mixed method post study | 1st year compulsory MBSR course participants: 140 students (Male = 64 (45 %); Female = 71 (53 %), 5 participants did not identify with a particular gender).  2nd-year optional course participants: 88 (Male = 39 (45 %), Female = 48 (55 %) 1 participant did not identify with a particular gender)  1st-year participants' ages ranges from 20 to 43 years (M = 24.4 years, SD = 3.4).  2nd-year participants' ages ranged from 22 to 37 years (Mage = 26.2 years, SD = 3.1). | Inclusion criteria for all participants was English language proficiency, as this was the primary language of the researchers and all questionnaires utilised in the study were designed for English speakers.  140 first-year students, 88 second-year students | All participants were given an open-ended qualitative feedback questionnaire with a satisfaction rating scale as well as a demographics questionnaire requesting gender, age, university course, and whether they had any previous experience of mindfulness. | MBSR adapted for medical students. The emphasis in the classes was on the experiential aspect of mindfulness practice. Practices included for example mindfulness of the breath, sitting meditation, choiceless awareness, body-scan, eating mindfully and walking mindfully. There was also some flexibility from session to session, depending on what issues arose for participants from week to week in their class. The course content for both first and second years was exactly the same albeit the duration of the sessions for second years was longer.  Duration: 7 weeks |
| Chung et al. 2018 US  Pre- and post-test pilot study | N=30  No control group  Age not reported  10 participants were excluded from the final analysis since they didn't complete all three survey (drop out rate 33%).  Demographic factors were not measured so it was not possible to determine whether the drop-outs were different from the students who completed all the surveys or not. | Students from three emergency medicine clerkship rotations during the summer. Students were selected to the clerkship.  No specific inclusion or exclusion criteria.  Third and fourth year students. | Self-reported behaviors and attitudes regarding meditation and mindfulness.  Overall reactions to the participation of the curriculum.  Likert scale (1-4) | A targeted mindfulness curriculum for medical students during their emergency medicine clerkship.  Duration: 4 weeks  Sessions included didactics about mindfulness, group discussions, role-plays and practice exercises in mindfulness and meditation.  Examples of the exercises: Breathing meditation, body scan, mindful eating (2 minutes each) |
| Danilewitz et al. 2016 Canada  Randomized controlled trial, pilot study,  WL controls | N= 30 (22 women) (n=15 intervention group, n=15 WL)  14 first year, 16 second year students  Drop out rate 27%  Analysed sample:  Intervention group (n=13) and WL group (n=9)  Age not reported | The first 30 who were interested  First and second year of medical studies | Primary outcome: Feasibility: ease of recruitment, program attendance and homework compliance.    Secondary outcomes:  DASS  the Jefferson Scale of Physician Empathy -Student Version  FFMQ  SCS  the Adapted Altruism Scale Satisfaction: A five-point Likert scale, ranging from Strongly Disagree (1) to Strongly Agree (5) | Peer-led mindfulness meditation program (MMP), an adapted version of MBSR.  Duration 8 weeks  Course themes address eg. work life balance, dealing with patient suffering, being mindful in clinical interactions, dealing with the need to be perfect, and self-acceptance.  Includes exercises, e.g body scan, awareness of breath, raisin exercise, loving kindness, sitting and walking meditation and yoga |
| Danilewitz et al. 2018 Canada  Prospective cohort study | 52 students (no control group) 36/52 (69,2%) were women.  Age: Mean age 23,8 years (+/- 2,7), range 20-37 years.  Drop out rate 28,8% | Inclusion: giving informed consent, understanding that the program is not a “therapy”.  No exclusion criteria.  The first 52 interested students were recruited  21 first-year, 20 second-year, 10 third-year students and one fourth-year student | Primary outcome, feasibility:  ease of recruitment, number of modules completed, satisfaction with the program, and adherence to a regular meditation practice.  Likert-type scales were used for many of the feasibility assessments as well as open-ended questions.  Secondary outcomes:  MBI  JSE-S  FFMQ-SF  SCS-SF | An online course: Mindfulness-Based Program for Medical Student Wellness, “MIND-MED”, video material and meditation practice. Information about the pillars of mindfulness practice and themes addressing the experience of medical students. Downloadable audio recordings and relevant reading material and links. Email reminders to the participants each week in case they hadn't completed the module. Formal practices such as body scan, sitting meditation, mindful yoga and walking meditation. Also informal exercises, such as mindful eating and mindfulness of daily activities.  Duration: 7weeks-4 months depending on the students progression |
| de Vibe et al. 2013  and  Solhaug et al. 2019  Norway  A randomized controlled trial | N=288  Intervention (n=144, 118 women), control group (n=144, 101 women).  176 (61%) of the sample were medical and 112 (39%) psychology students  Intervention group: 86 (60%) medical students,  Control group: 90 (62%) medical students  Mean age 23,8 years (SD=5,2), intervention 23,6 (SD=4,7) and control 24 (SD=5,7)  6 students did not turn up for the intervention, 6 discontinued intervention and 11 students were lost to follow-up. No significant differences between the drop-outs and the rest of the participants. | Inclusion: medical and psychology students in their second or third term at the time of the recruitment in the two Norwegian universities.  No exclusion criteria.  Second or third term | GHQ12  MBI  PMSS  SWB  FFMQ  The compliance was measured by recording the number of classes attended as well as the amount of home practice | Mindfulness-based stress reduction (MBSR)  Duration: 7 weeks, 4 years (Solhaug et al. 2019) and 6 years (deVibe et al. 2018)  Included physical and mental mindfulness exercises.  In addition, the course had theory about mindfulness, stress, stress management and mindful communication. Reflections on practising mindfulness were shared in a group.  The intervention group was offered 90-min booster sessions semi-annually during the 6-year follow-up. |
| Erogul et al. 2014  US  Prospective, unblinded, randomized controlled study | N=59  2/59 (3%) dropped out and fine N=57 (26 women)  intervention group (n= 28, 12 women)  control group (n=29, 14 women)  Mean age 23,5 years (SD=1,7). In control group 23,3 (1,4) and in the intervention group 23,6 (1,9) | Participants were selected at random form a first-year class of medical student.  No inclusion or exclusion criteria were mentioned.  First-year students | PSS (10-question version)  SCS (26-question version)  RS(14-question version) | Abridged MBSR course.  Duration: 8 weeks and one full-day retreat  The sessions had both theory about understanding stress (cognitive curriculum) and practice in mindfulness meditation(experiential part). The experiential practices included for example body scan and breathing-based yoga. Participants could not miss more than 1/8 sessions and they had to present at the retreat. |
| Garneau et al. 2013  Canada  A pre- and post-test study | 58 students, 74% were women Age: Average age 26 years | Inclusion criteria: 4th-year students choosing an elective.  Exclusion criteria not reported.  4th-year medical students | MBI-HSS  PSS-10  SPWB  SCS  MAAS  BDI-II  In addition, Students rated the course on a scale of 0 to 10 in terms of its value for them, with 10 very important.  They also indicated to what extent they viewed various aspects of the course as useful and beneficial (on a 1 to 10 scale, with 1 = not useful at all). | Mindful Medical Practice, modeled after MBSR but has more emphasize on communication (e.g. role plays). Practices include for example body-scan, sitting meditation, yoga, loving kindness and meditation involving imagery.  Duration: 4 weeks |
| Greeson et al. 2015  US  A prospective, observational, and mixed methods design | A total of 44 students, 29 of them women. Age not reported.  Drop out rate 18% | All students in the medical school were eligible to participate  33 first-year, one second-year, three third-year, five fourth-year, and two MD/PhD students | CAMSR  PSS  Feasibility, (rates of workshop enrollment and completion), | A mind-body course. The sessions included instructor-led meditation, sharing experiences, didactic on the science of mind-body medicine and practicing mind-body skills. Practiced skills included for example mindfulness meditation, relaxation breathing, guided imagery, creative self-expression through drawing, body awareness, progressive muscle relaxation, mindful eating, and a loving kindness meditation. The sessions ended with reding of a poem or a passage related to mindfulness or self-care.  Duration: 4 weeks |
| Keng et al. 2015  Malaysia  A controlled study | 139 students  intervention group (n=82, drop out rate 7% i.e. final sample 77)  control group (n=57, no drop-outs)  61.2% (n = 82) were women.  Age: Mean 22.4 years (SD =0.55, range 21–24) | Inclusion: fourth -year students in their psychiatric rotation.  Exclusion: students who reported to be suicidal | MAAS  PSS  DASS-21  GHQ-12  SHS  SWLS | Mindful -Gym (based on MBSR and MBCT) (Phang et al. 2015a). Includes elements of cognitive behaviour therapy (identification of cognitive distortions and progressive muscle relaxation). Mindfulness exercises: mindful breathing, mindful stretching, body scan meditation, and mindful imagery  Duration: 4 weeks |
| Kuhlmann et al. 2016 Germany  A prospective randomized controlled trial (three-arm) | Allocated at baseline : 66 students in MediMind, 73 in Autogenic training and 43 in control group.  Follow-up sample:  35 students in MediMind, 42 in Autogenic training and 22 in control group.  Post test sample:  31 students in MediMind, 32 in Autogenic training and 17 in control group.  67 (84%) women  Mean age 23,3 years in MediMind, 23,7 years in AT and 22,9 years in control group. | Inclusion: medical students of 2nd and 8th semester, dental students of 2nd semester.  Medical students of second and eight semester and dental students of second semester (because the preclinical studies are almost the same for both).  48 (60%) preclinical students and 32 (40%) clinical students. | Primary outcomes:  TIC  Brief COPE  Secondary outcome:  BSI | Intervention group attended Medi-Mind (MM) course, mindfulness-based stress prevention training for medical students. Standard treatment group attended Autogenic training (AT), an auto-suggestive relaxation technique, where the participants learned how to instruct themselves to suggest specific autonomic sensations such as muscular relaxation, vascular dilatation, stabilization of heart function or regulation of breathing).  MediMind structure: various types of mindfulness meditation; themes such as "What is mindfulness?", ‘Satellite-position’ as a target state of successful stress management (the ability to observe one’s thoughts, emotions, physical reactions and impulse to act), learning to address intrusive and distracting thoughts or feelings, dysfunctional cognitive judgement mechanisms, personal standards and assumptions, and stress-tolerance skills and the concept of radical acceptance.  Duration: 5 weeks and 1 year follow-up |
| Malpass et al. 2019  UK  A qualitative study | 57 students, 15 were male. Age not reported | Inclusion and exclusion criteria not presented.  8 first-year students, 15 second-year students, 17 third-year students, 7 fourth-year students, 3 fifth-year students and 4 unknown | A free text survey at the end of their mindfulness course.  In addition, six qualitative interviews were conducted lasting between 60 and 90 minutes. Interviews used a topic guide. | Course content followed the manualised curriculum of “Mindfulness-Based Cognitive therapy”. Instruction consists of various formal and informal meditation practices, including guided body scans, sitting and walking meditations, mindful movement (based on Hatha yoga), 3-minute breathing spaces, and focused awareness on routine daily activities.  Duration: 8 weeks |
| Moir et al. 2016 New Zealand  Randomized controlled trial | N=275 (145 women)  intervention group (n=133, 68 women), control group (n=142, 77 women)  Mean age 20,9 years (SD=2,9) in the total population  Controls mean 20,9 years (2,6), intervention mean 20,8 years (3,1)  232/275 (84%) completed the study.  Lost to follow-up:  22/133 (17%) in intervention  21/142 (15%) in control group | Inclusion: second- or third-year students.  Exclusion: already trained as a peer leaders  Second- and third-year students (148 second-year and 127 third-year students) | Primary outcomes: PHQ-9  GAD-7  Secondary outcomes: LASA Resilience: a 25-item resilience questionnaire  Academic self-concept: Perceived Competence Scale Academic motivation: the Motivated Strategies for Learning Questionnaire | A Peer-led program of peer support and peer-taught mindfulness practice.  Duration:23 weeks (semester breaks between)  Included peer-led mindfulness sessions, two social gatherings and the possibility to approach the peer leader for support.    Mindfulness sessions were not described to detail. |
| Moore et al. 2020 Australia  A single-arm prospective mixed method cohort study | N=47 (38 women)  No controls  Mean age at baseline 26,7 years (SD=3,9)  Drop outs:  8 weeks: 13/47 (28%)  4 months: 19/47 (40%)  Final sample size:  8 weeks n=34  4 months n=28  Students who finished the 8-week program and submitted their weekly practice data did not statistically differ from non-completers regarding the baseline characteristics. | Inclusion: a penultimate-year student.  No exclusion criteria mentioned.  Penultimate-year students | Feasibility was measured as the proportion of participants who still practised formal mindfulness by the conclusion of MTP, and the frequency and duration of practice.  Effectiveness:  PSS  SCS  CS  also 500-word reflective essay | Online Mindfulness Training Programme (MTP)  Duration: 8 weeks and follow-up of 4 months  8x10min weekly mini-lectures (audiovisual recording via email) and daily (Monday to Friday) 5 min guided mindfulness meditation sessions (audio recording).  The lecture contents eg. about multi-tasking, stress reduction, distraction and procrastination, mindful communication, emotion regulation, compassion (towards self and others) and mindful use of technology.  Meditation sessions included eg. body scan, mindful breathing, thought-labelling, mindful listening, working mindfully with emotions, loving-kindness, self-compassion and mountain meditation |
| Neto et al. 2019 Brazil  Randomized controlled trial | N=141  Intervention (n=70, 36 women), control (n=71, 35 women)  Mean age: Intervention group 18,9 years (SD=1,8) control group 19,1 years (SD=1,96)  Drop outs: all 141 participants included in intention- to-treat-analysis (19% did not answer the post-test questionnaire) | Inclusion: at least 18 years old and enrolled in the first year of medical school.  Exclusion: not completing all the questionnaires, not signing the consent form, withdrewing from medical school, absence at the time of data collection  First-year students | WHOQOL-Brief  DASS 21  FFMQ-BR | A large-group (n=45) mindfulness meditation course (based on the methods of Kabat-Zinn).  Duration: 6 weeks  The sessions were practical including lots of meditation and discussion.  Mindfulness practices included eg. raisin exercise, body scan, nonjudgemental listening, mountain meditation, mindfulness of breathing, walking meditation, sitting meditation and loving kindness.  Control group attended a course about organizational aspects of medical school. |
| Phang et al. 2015a  Malaysia  Randomized controlled trial | 75 in total  intervention group (n=37)  control group (n=38)  Intervention group 26 (70%) females  Control group 31 (82%) female  Mean age:  intervention group 21,14 (SD=1,10) years  control group 20,94 (SD=1,17) years  Drop outs: all 37 participants included in intention- to-treat-analysis (one control did not answer the post-test questionnaire) | Interested students were included. If students weren't ready to attend at least 80% of the sessions and spend 3-5 minutes on practice daily, they were excluded.  Intervention group: 10 (27%) 1st-year students, 14 (38%) 2nd-year students and 13 (35%) 3rd-year students.  Control group: 12 (32%), 11 (29%) and 15 (40%), respectively. | MAAS  PSS  GHQ  GSE  Compliance with the program | Mindfulness-based stress management/Mindful Gym. Based on MBSR and MBCT but having more emphasis on informal practice. Sessions had both theory and practice and included for example practice of loving-kindness, gratitude and instructions for medical students. Examples of exercises: mindful stretching and muscle relaxation, mindful breathing, mindful imaginary, body scan and kindness and STOP  Duration: 5 weeks, follow-up 6 months |
| Phang et al. 2015b Malaysia  A single-group, prospective study | A total of 135 students.  84 (62 %) females and 51 (38 %) males  Age: M=22.23, (SD)=0.64 years (range 21-25 years)  Drop outs: 4% | Inclusion criteria: year-four medical students doing psychiatric posting and could commit to at least three out of the four sessions in the program.  Exclusion criteria: students who had participated in mindfulness training  Year-four medical students doing psychiatric posting | PSS-10  GHQ-12  MAAS  In addition a questionnaire was used to obtain feedback on the intervention program with regards to whether the program was easy to understand, practical, beneficial, and applicable to patient care. | Brief Group Mindfulness-based Cognitive Therapy (b-GMBCT), Mindful Gym. For example: mindful stretching and muscle relaxation (week 1), deep and mindful breathing and imagery (week 2), Mindful S.T.O.P. and grateful thinking (week 3), and body scan and kindness (week 4). The program was delivered using both didactic and experiential approaches  Duration: 4 weeks |
| Phang et al. 2015c and  Kar et al. 2015 Malaysia  A randomized controlled study | 76 students, 38 in the intervention and 38 in the control group.  Intervention group, 76% (29) female; control group 82% (31) female.  Mean age in the intervention group was 20,91 (1,15) years and 20,94 (1,17) years in the control group.  Drop outs: all 76 participants included in intention- to-treat-analysis (one control did not answer the post-test questionnaire) | Inclusion: 1st to 3rd-year students. Exclusion: could not commit to the program (3-5 min daily home practice)  1-3 year students. In the intervention group, there were 34% 1st-year students, 34% 2nd-year students and 32% 3rd-year students. In the control group, the same numbers were 32%, 29% and 39%. | MAAS  PSS  DASS  GSE  In addition, a one-item questionnaire was constructed to assess compliance with weekly practice and another question was constructed to obtain feedback on the most frequently used stress reduction tool among the participants | Mindful-Gym DVD, based on the principles of the eight-week MBSR and MBCT programs. It is shorter in duration with more emphasis on informal practice, has sessions on grateful thinking and cultivating loving-kindness; and with instructions tailored for medical students. The DVD contained about 500 PowerPoint slides with step-by-step audio-video instructions for coping with stress. The training materials were organized in five modules (one for each week). Participants were taught techniques to focus their attention on the present moment experience, decrease emotional reactivity, and increase feelings of gratitude.  Duration: 5 weeks |
| Rosenzweig et al. 2003  US  Prospective, nonrandomized, cohort-controlled trial | Intervention group (n=140)  Control group (n=162)  Female-male ratio not reported.  Approximately 40 students (18% of the 2nd-year students) participated the course each year.  Age not reported.  Drop outs:  Intervention group 11%  Control group 6% | Inclusion: second-year students  Exclusion: none mentioned | POMS | MBSR course, included for example the following exercises: body scan, breath awareness, mindful stretching (Hatha Yoga), eating meditation, walking meditation, and guided imagery (mountain/lake meditations).  Control group participated in didactic sessions about complementary medicine (sessions included also demonstrations, group discussions and student presentations)  Duration: 10 weeks |
| Shapiro et al. 1998  US  A matched randomized experiment, wait-list control design | 78 students randomized to intervention (n=39) and control group (n=39)  Drop out: 1 in intervention group, 4 in control group  Final sample n= 73 students:  Intervention n=38, control n=35  32 males and 41 females  Age not reported. | Inclusion: apparently premedical, first-year and second-year medical students.  Exclusion: not willing to be assigned to either the intervention or the control group  35 premedical and 38 medical students | ECRS  SCL-90-R for distress, Subscale 4 of the SCL-90 for depression  STAI Form 1  INSPIRIT  Also compliance (diaries) and course evaluation were collected | The course was a modeled version of the Stress Reduction and Relaxation Program (SR&RP) developed by Kabat-Zinn. Different practices included sitting meditation, body scan, hatha yoga, loving kindness and forgiveness meditation. In addition, there were exercises on mindful listening skills and empathy as well as didactic material on stress.  Duration: 8 weeks |
| Shapiro et al. 2019  US  A randomized controlled trial | 41 students, 20 in the full intervention group, 21 students attended only the introductory class (control group).  32 females (intervention 14 females, control 18 females)  Average age in the intervention group 23,9 years and in the control group 25,1 years  Drop outs: not reported | Apparently all who requested to participate were included.  Preclinical, 13 first- and 28 second -year students. | FFMQ  PSS  PHQ-8  JSE  a Likert-type educational outcomes survey  In addition, students were asked three open-ended questions: What advantages/disadvantages mindfulness has over other therapies for treating mental health problems? What do you think you can get out of this course? What are some problems that you have had/anticipate having with taking a mindfulness course? | The introductory session before the course included theory about mindfulness and some exercises (guided sitting meditation, walking meditation, and mindful yoga). The course was an adapted version of the MBSR. Each session had guided sitting and walking meditation.  Duration: 8 weeks |
| van Dijk et al. 2015, 2017  the Netherlands  A cluster-randomized controlled trial | N=167  Intervention (n=83, 60 women) "clerkship as usual" (CAU) group (n=84, 71 women).  Participants were cluster-randomized by their clerkships groups.  Mean age:  CAU 23,3 years (SD=1,8),  Intervention 23,7 years (SD=1,9)  Drop outs:  all 167 participants included in intention- to-treat-analysis (one control did not answer the post-test questionnaire)  Examples of drop outs after allocation:  Three months follow-up:  Intervention group 10/83 (12%) persons (of whom 3 persons did not start the course); control group 16/84 (19%)  Twenty months follow-up: Intervention group 16/83 (19%); control group 26/84 (31%) | Inclusion: students starting their neurology clerkships during the recruitment period.  Exclusion: under 18 years of age, non-Dutch speaking and previous participation in MBSR  Students in their first year of clinical clerkship | Primary outcome: BSI  Secondary outcomes:  MHC-SF  LiSat-9  JSPE  FFMQ  IBI  Home practice was recorded through a Likert-type scale. | Mindfulness-based stress reduction (MBSR) including some alterations.  Duration: 8 weeks, and follow-ups after 3, 7, 12, 15 and 20 months  Weekly session themes were for example Recognizing Automatic Behavior, Influence of Perception, Recognizing Boundaries, Awareness of Stress, Communication and Work–Life Balance  Mindfulness practices included for example raisin practice, body scan, sitting meditation, yoga, and walking meditation. |
| Warnecke et al. 2011 Tasmania  Single‐blinded, randomised controlled multi-center trial | N=65 (42 women)  intervention (n=31, 23 women), control (n=34, 19 women)  Mean (SD) age years: n=65: 23,9 (3,2).  Intervention group n=31: 23,4 (2,1)  Control group n=34: 24,4 (4,0).  Lost to follow-up:  At 8 weeks: intervention group (n=7), control group (n=2)  At 16 weeks: intervention group (n=5) | Inclusion: medical students in their final 2 years (in three clinical schools).  Exclusion: participation in the pilot trial or a K10 questionnare score of 30 or more (high psychological distress and high risk for depression and anxiety).  Final 2 years of studies | Primary outcome:  PSS  Secondary outcome:  DASS | CD-guided spoken mindfulness practice of duration of 30 minutes designed for the study. The participants were asked to follow the practice independently during each day over a period of 8 weeks.  Duration: 8 weeks and for the intervention group additional 16 weeks follow-up |
| Weingartner et al. 2019 US  Pre- and post-test, mixed methods design | N=45, during three years (2015-2017)  No controls  Female-male ratio not reported  Age not reported  2015-2016 courses:  11/25 (44%) of the participants completed both KIMS-surveys.  2017 course (shorter):  16/20 (80%) completed both KIMS-surveys.  38/45 (84%) provided qualitative data (course evaluations and/or the post-course survey). | 2015 course: pre-clinical second-year students  2016-2017 courses: fourth- and second-year students  Second- and fourth year students (20 second-year and 25 fourth-year students). | KIMS  open-ended questionnare for subjective feedback (course experience, sustained use of skills, perceptions of utility, benefits and teansferrable skills) | Compassion Cultivation Training (CCT).  Duration in 2015-2016 courses: 8 weeks  Duration in 2017 course: 5 weeks  Weekly sessions included pedagogical instruction, group discussion, guided meditation in a group (for example loving kindness), mindfulness training, listening and communicating exercises and practical exercises linked to weekly changing compassion themes. |
| Yang et al. 2018  US  A prospective, randomized controlled trial | 88 students, 45 in the intervention and 43 in the control group.  32 (36%) were male and 56 (64%) were female  Mean age was 25,11 years (range 21-47) | Inclusion: all the students of the school. Exclusion: no smartphone  26 (29.5%) First-year, 32 (36.4%) second-year, 15 (17.0%) third-year and 15 (17.0%) fourth-year students. | PSS  FFMQ  GWBS  Additional open ended questions, to evaluate sources of stress, coping strategies, and current and past exposure to meditation and other forms of alternative therapies. | The mobile application Headspace. The mindfulness program has a following structure: for the first 10 days the sessions last 10 minutes, next 15 days 15 minutes and 20 minutes for the rest of the days. The sessions consist of audio-guided instructions, such as taking notice of the body while seated comfortably, different breathing exercises, sensing emotions and acknowledging thoughts.  Duration: 30 days and follow-up 60 days |

**Abbreviations:**

Beck Depression Inventory II (BDI-II); Brief Symptom Inventory (BSI); Cognitive and Affective Mindfulness Scale—Revised (CAMSR); The Compassion Scale (CS); The Five Facets of Mindfulness Questionnaire (FFMQ); the Five Face of Mindfulness Questionnaire-short form (FFMQ-SF),; the Depression, Anxiety and stress scale (DASS); Empathy Construct Rating Scale (ECRS); Generalized Anxiety Disorder questionnaire (GAD-7); General Health Questionnaire (GHQ); General Self-efficacy Scale (GSE); General Well-Being Schedule (GWBS); Irrational Beliefs Inventory (IBI); The Index of Core Spiritual Experiences (INSPIRIT); the Jefferson Scale of Empathy-medical student version (JSE-S); Jefferson Scale of Physician Empathy (JSPE); the Kentucky Inventory of Mindfulness Skills (KIMS); Quality of life: Linear Analogue Self-Assessment (LASA); Life Satisfaction Questionnaire (LiSat-9); Mental Health Continuum-Short Form (MHC-SF); Mindful Awareness Attention Scale (MAAS); Maslach Burnout Inventory (MBI); Maslach Burnout Inventory-Human Services Survey (MBI-HSS); Patient Health Questionnaire (PHQ-9); the Personal Health Questionnaire (PHQ-8); Perceived Medical School Stress (PMSS); the Perceived Stress Scale (PSS); Profile of Mood States (POMS); the Resilience Scale (RS); The Hopkins Symptom Checklist 90-Revised (SCL-90-R); Satisfaction With Life Scale (SWLS); the Self-Compassion Scale (SCS); the Self Compassion Scale-short form (SCS-SF); Subjective Happiness Scale (SHS); Scales of Psychological Well-Being (SPWB);The State-Trait Anxiety Inventory (STAI); Subjective Well-Being scale (SWB); Trier Inventory for the Assessment of Chronic Stress (TICS); World Health Organization Quality of Life (WHOQOL-Brief)

**References:**

van Dijk, I., Lucassen, P. L., & Speckens, A. E. (2015). Mindfulness training for medical students in their clinical clerkships: Two cross sectional studies exploring interest and participation. BMC Medical Education, 15(1), 24.

Other references, please see manuscript list of references.

**Supplemental Digital Content Table 4a.** the Medical Education Research Study Quality Instrument (MERSQI) rating

|  | Aherne et al. 2016 | Chung et al. 2018 | Danilewitz et al. 2016 | Danilewitz et al. 2018 | de Vibe et al. 2013, Solhaug et al. 2019 | Erogul et al. 2014 | Garneau et al. 2013 | Greeson et al. 2015 | Keng et al. 2015 | Kuhlmann et al. 2016 | Moir et al. 2016 | Moore et al. 2020 |
| --- | --- | --- | --- | --- | --- | --- | --- | --- | --- | --- | --- | --- |
| **Study design** |  |  |  |  |  |  |  |  |  |  |  |  |
| 1. Study design | 1 | 1,5 | 3 | 1,5 | 3 | 3 | 1,5 | 1,5 | 2 | 3 | 3 | 1,5 |
| **Sampling** |  |  |  |  |  |  |  |  |  |  |  |  |
| 2. No. of institutions studied | 0,5 | 0,5 | 0,5 | 0,5 | 1 | 0,5 | 0,5 | 0,5 | 0,5 | 0,5 | 0,5 | 0,5 |
| **Response rate** |  |  |  |  |  |  |  |  |  |  |  |  |
| 3. Response rate, %* | 0,5 | 1 | 1 | 1 | 1,5 | 1,5 | 0,5 | 1,5 | 1,5 | 1 | 1,5 | 1 |
| **Type of data** |  |  |  |  |  |  |  |  |  |  |  |  |
| 4. Type of data | 1 | 1 | 1 | 1 | 1 | 1 | 1 | 1 | 1 | 1 | 1 | 1 |
| **Validity of evaluation instrument** |  |  |  |  |  |  |  |  |  |  |  |  |
| 5. Internal structure | 0 | 0 | 1 | 1 | 1 | 1 | 1 | 1 | 1 | 1 | 1 | 1 |
| 6. Content | 0 | 0 | 1 | 1 | 1 | 1 | 1 | 1 | 1 | 1 | 1 | 1 |
| 7. Relationships to other variables | 0 | 0 | 0 | 0 | 0 | 0 | 0 | 1 | 1 | 0 | 0 | 0 |
| **Data analysis** |  |  |  |  |  |  |  |  |  |  |  |  |
| 8. Appropriateness of analysis | 1 | 1 | 1 | 1 | 1 | 1 | 1 | 1 | 1 | 1 | 1 | 1 |
| 9. Complexity of analysis | 1 | 2 | 2 | 2 | 2 | 2 | 2 | 2 | 2 | 2 | 2 | 2 |
| **Outcomes** |  |  |  |  |  |  |  |  |  |  |  |  |
| 10. Outcomes | 1 | 2 | 2 | 2 | 2 | 2 | 2 | 2 | 2 | 2 | 2 | 2 |
|  |  |  |  |  |  |  |  |  |  |  |  |  |
| **Total score** | 6 | 9 | 9,5 | 11 | 10,5 | 10 | 10,5 | 12,5 | 11 | 9,5 | 10 | 9,5 |
| * based on response rate at post-test, not at potential later follow-ups | | | | |  |  |  |  |  |  |  |  |

|  | Neto et al. 2019 | Phang et al. 2015a | Phang et al. 2015b | Phang et al. 2015c | Rosenzweig et al. 2003 | Shapiro et al. 1998 | Shapiro et al. 2019 | van Dijk et al. 2015, 2017 | Warnecke et al. 2011 | Weingartner et al. 2019 | Yang et al. 2018 |
| --- | --- | --- | --- | --- | --- | --- | --- | --- | --- | --- | --- |
| **Study design** |  |  |  |  |  |  |  |  |  |  |  |
| 1. Study design | 3 | 3 | 1,5 | 3 | 2 | 3 | 3 | 3 | 3 | 1,5 | 3 |
| **Sampling** |  |  |  |  |  |  |  |  |  |  |  |
| 2. No. of institutions studied | 0,5 | 0,5 | 0,5 | 0,5 | 0,5 | 0,5 | 0,5 | 0,5 | 1,5 | 0,5 | 0,5 |
| **Response rate** |  |  |  |  |  |  |  |  |  |  |  |
| 3. Response rate, %* | 1,5 | 1,5 | 1,5 | 1,5 | 1,5 | 1,5 | 0,5 | 1,5 | 1,5 | 1 | 1,5 |
| **Type of data** |  |  |  |  |  |  |  |  |  |  |  |
| 4. Type of data | 1 | 1 | 1 | 1 | 1 | 1 | 1 | 1 | 1 | 1 | 1 |
| **Validity of evaluation instrument** |  |  |  |  |  |  |  |  |  |  |  |
| 5. Internal structure | 1 | 1 | 1 | 1 | 1 | 1 | 1 | 1 | 1 | 1 | 1 |
| 6. Content | 1 | 1 | 1 | 1 | 1 | 1 | 1 | 1 | 1 | 1 | 1 |
| 7. Relationships to other variables | 1 | 0 | 1 | 0 | 0 | 1 | 1 | 0 | 0 | 0 | 0 |
| **Data analysis** |  |  |  |  |  |  |  |  |  |  |  |
| 8. Appropriateness of analysis | 1 | 1 | 1 | 1 | 1 | 1 | 0 | 1 | 0 | 1 | 1 |
| 9. Complexity of analysis | 2 | 2 | 2 | 2 | 2 | 2 | 1 | 2 | 2 | 2 | 2 |
| **Outcomes** |  |  |  |  |  |  |  |  |  |  |  |
| 10. Outcomes | 2 | 2 | 2 | 2 | 2 | 2 | 2 | 2 | 2 | 2 | 2 |
|  |  |  |  |  |  |  |  |  |  |  |  |
| **Total score** | 11 | 10 | 11 | 10 | 10 | 11 | 8 | 10 | 10 | 9,5 | 10 |
| * based on response rate at post-test, not at potential later follow-ups | | | | |  |  |  |  |  |  |  |

**Supplemental Digital Content Table 4b.** JBI Critical Appraisal Tool

|  | Malpass et al. 2019 | Aherne et al. 2016 | Moore et al. 2020 | Weingartner et al. 2019 | Garneau et al. 2013 | Greeson et al. 2015 |
| --- | --- | --- | --- | --- | --- | --- |
| 1.       Is there congruity between the stated philosophical perspective and the research methodology? | unclear | no | no | yes | no | no |
| 2.       Is there congruity between the research methodology and the research question or objectives? | yes | yes | yes | yes | yes | yes |
| 3.       Is there congruity between the research methodology and the methods used to collect data? | yes | yes | yes | yes | yes | yes |
| 4.       Is there congruity between the research methodology and the representation and analysis of data? | yes | yes | yes | yes | no | yes |
| 5.       Is there congruity between the research methodology and the interpretation of results? | yes | yes | yes | yes | no | yes |
| 6.       Is there a statement locating the researcher culturally or theoretically? | no | no | no | yes | no | no |
| 7.       Is the influence of the researcher on the research, and vice- versa, addressed? | yes | no | no | no | no | no |
| 8.       Are participants, and their voices, adequately represented? | yes | yes | yes | yes | no | yes |
| 9.       Is the research ethical according to current criteria or, for recent studies, and is there evidence of ethical approval by an appropriate body? | yes | yes | yes | yes | yes | yes |
| 10.    Do the conclusions drawn in the research report flow from the analysis, or interpretation, of the data? | yes | yes | unclear | yes | unclear | unclear |

**Supplemental Digital Content Figures.** Forest plots of effect sizes of mindfulness interventions on different outcomes.


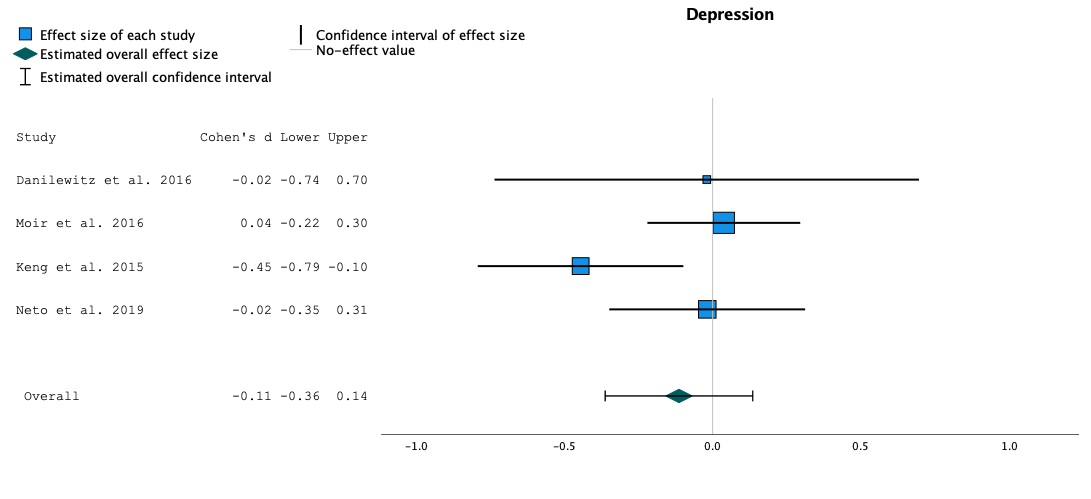


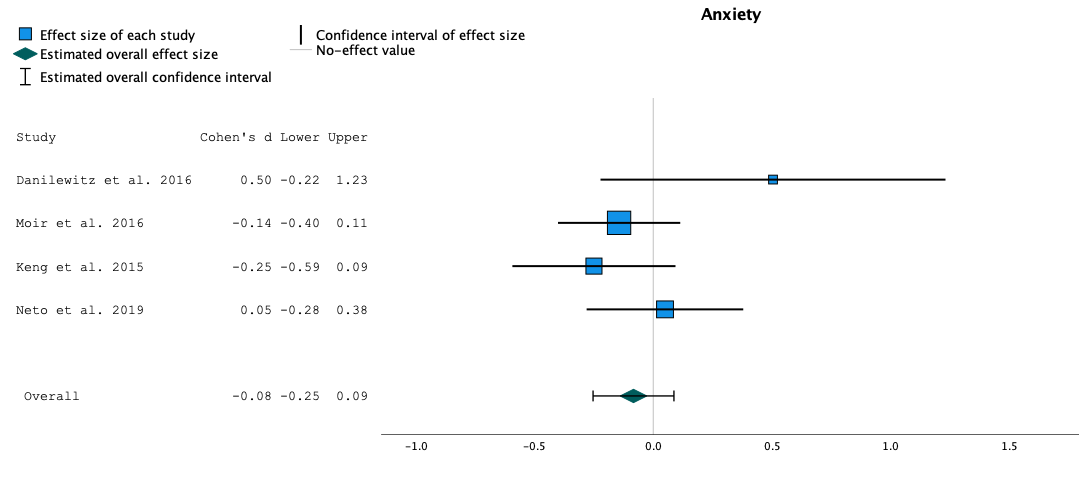


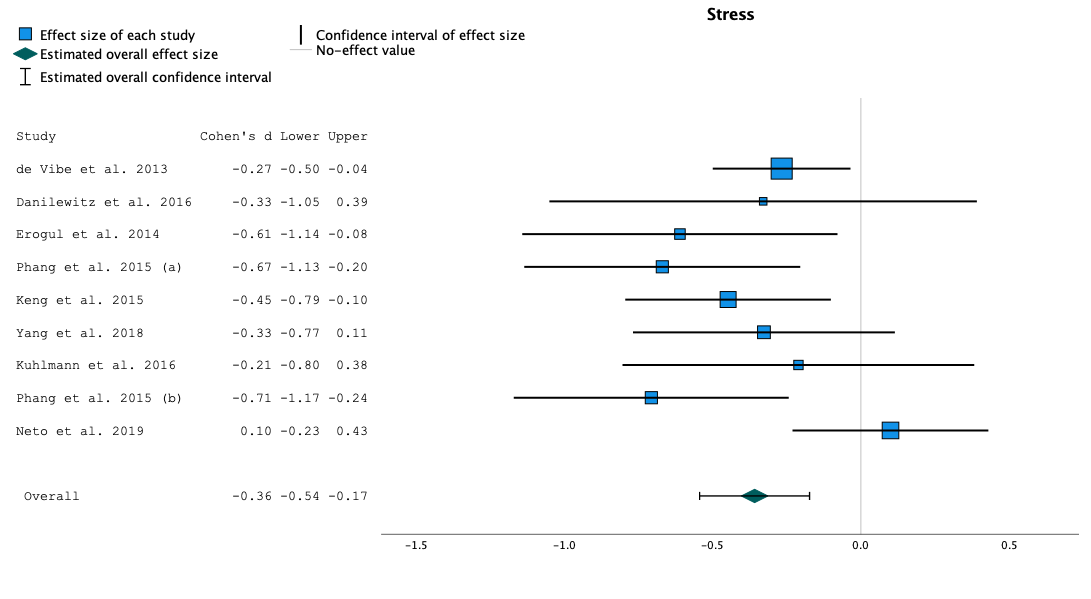


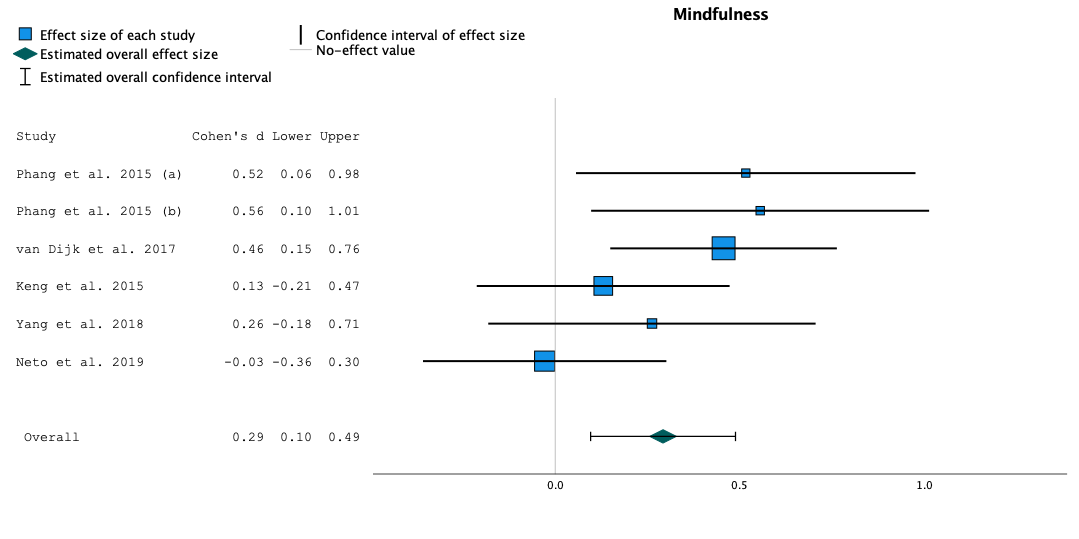


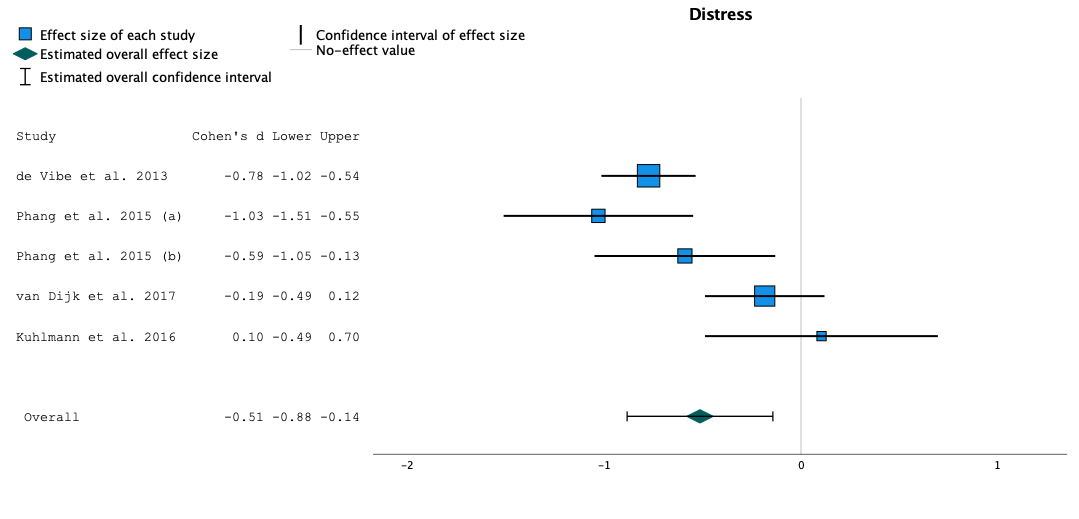


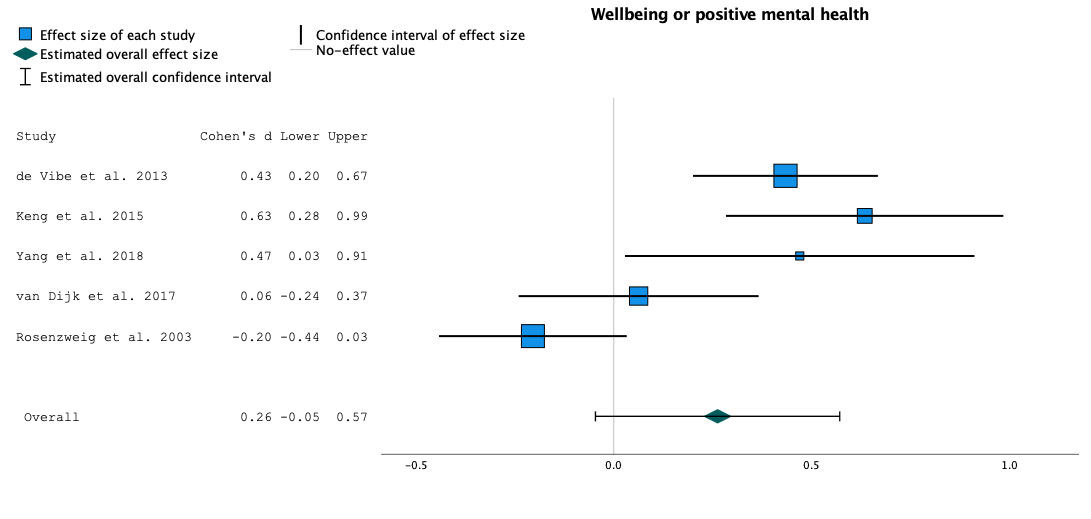


**Supplemental Digital Content.** Qualitative results

In summary, six broad themes were identified: Increased awareness and mindfulness; Increased well-being, resilience and balance; Kindness and compassion towards others and self; Work or studies related benefits; Factors affecting feasibility and satisfaction; and Difficulties and adverse effects.

**Increased awareness and mindfulness**

After mindfulness intervention, increased awareness was constantly reported, towards oneself (e.g. Greeson et al. 2015), others (Garneau et al. 2013), own thoughts (Malpass et al. 2019, Moore et al. 2020), emotions and body (e.g. Moore et al. 2020), and behavior (e.g. Moore et al. 2020). *“The course enabled me to be more aware of myself, my colleagues, my patients and my limitations.”* (Garneau et al. 2013). *“The course helped me reflect on my behavioural patterns and thinking processes.”* (Garneau et al. 2013). As concluded by Moore et al. (2020) “Participants described an increased awareness of the nature of the mind, including mind-wandering, the arising of emotions and habitual reactions to these emotions, self-criticism, stress and anxiety about doing things correctly, judgment of others, procrastination and constant thinking and analysis.”

Greater awareness resulted also to earlier recognition of stress and better possibilities to manage stress and regulate own behavior. *“I now notice more when I’m getting stressed and try and do something about it rather than just letting it be there and I am better at knowing what kind of things stress me out.”* (Malpass et al. 2019). It created a possibility to have space between stimulus and response, *“Through becoming more aware of the emotions I am experiencing, I have been able to recognize how my body is responding to these emotions... In recognising when I am stressed or experiencing an emotion... I am able to stop myself, acknowledge that I’m feeling that way and accept the emotion without converting it to an unwanted action”* (Moore et al. 2020).

Increased awareness of negative thoughts, in a non-judgmental way, was important for building a new relationship with own thoughts and feelings, *“… I have become more aware of acknowledging my thoughts in a state of non-judgemental awareness. I am now acknowledging my thoughts and choosing to redirect my focus. No longer do I feel annoyed with myself if these thoughts return, I simply acknowledge them and refocus”* (Moore et al. 2020).

The participants also noticed how awareness may help in clinical work, *“I think becoming more aware of how I think about things has made me more aware of how other people might think about things, I guess that would make me a better practitioner.”* (Malpass et al. 2019).

There was also some experiences of enhanced awareness towards own body, *“Now, whenever I feel overwhelmed I make a conscious effort to listen to what my body is telling me.”* (Garneau et al. 2013). This led to change of habits, *“We were encouraged to trust and listen to our bodies rather than tune them out (for example, when tired - continue working, when sick - go to work anyway).”* (Garneau et al. 2013).

**Increased well-being, resilience and balance**

Participants reporter decreased anxiety and less depression (Weingartner et al. 2019), decreased stress (Greeson et al. 2015, Moore et al. 2020) and less tiredness (Weingartner et al. 2019). They also reported less judgement (Moore et al. 2020), decreased comparing to other (Malpass et al. 2019), increased acceptance (Garneau et al. 2013, Malpass et al. 2019, Moore et al. 2020), concentration (Malpass et al. 2019), patience (Weingartner et al. 2019), and happiness (Malpass et al. 2019, Weingartner et al. 2019). They felt better sense of balance (Greeson et al. 2015) and control, resilience and flexibility, and better ways of coping (Malpass et al. 2019) and managing stress (Greeson et al. 2015). *“I have learned to be a better and more balanced ‘me’.”* (Greeson et al. 2015). *“I feel more in control now, like I have a resource to fall back on, something to help me.”* (Malpass et al. 2019).

Learning mindfulness had led to change in experience, attitude and life, *“its changed the way I experience the world and my own emotions. Set me on a path towards healthy processes”* (Malpass et al. 2019), *“the course has changed my mindset, how I think of myself, how I react to situations,”* (Malpass et al. 2019). *“I have been working towards pausing before reacting and understanding that emotions are transient, but the impact of our behaviour as a result of these emotions can have a lasting effect. This change in attitude and behaviour has allowed me to remain calm when in situations that are potentially volatile.”* (Moore et al. 2020).

**Kindness and compassion towards others and self**

Participants of mindfulness interventions learned means of and appreciation of self-care, *“there are many aspects of self- care.”* (Greeson et al. 2015), *“The course made me think about how to look after myself better”* (Malpass et al. 2019), *“learn means of taking care of one self”* (Garneau et al. 2013). They reported increased self-compassion, *“If I don’t get work done I don’t beat myself up about it. Mindfulness teaches me to be kinder to myself.”* (Malpass et al. 2019). *“… helped me look at my situation objectively and think about how I would judge someone else in the same circumstances. This made me more forgiving of how I was performing, which in turn made me aware of how tiring and self-defeating it was to be expending energy on self- criticism...”* (Moore et al. 2020).

Learning of acceptance was important factor relating to self-compassion, *“I was very judgemental with myself and was striving towards an idea of perfection that I couldn’t achieve. I’ve noticed I am a lot more accepting of my imperfections . .” “Well I think accepting that things are difficult. You can’t do everything ... everyone finds things difficult and it’s okay to find it difficult. So I think I took that [judgement] away.”*(Malpass et al. 2019). “No longer do I feel annoyed with myself if these thoughts return, I simply acknowledge them and refocus” (Moore et al. 2020).

Enhanced compassion in general, and especially towards patients was reported. Based on Moore et al. (2020) participants’ increased awareness of “I’m too busy” self-talk allowed them to make more time to help others. *“[I learned] how to be compassionate and caring without taking on the patient’s pain myself.”* (Weingartner et al. 2019), *“the loving kindness meditation helped “to identify personal judgement towards patients” and consciously “maintain compassion towards them”* (Moore et al 2020).

**Work or studies related benefits**

Increased concentration and efficiency (Malpass et al. 2019) was experienced. Increased awareness and via this change in working habits was related to better efficiency, *“I just recognise when I’ve got to that point where I’m not going to take anything else in, where I just need some time out to go away and come back ... in terms of workload it’s just helped me be more efficient in my studies.”* (Malpass et al. 2019). *“I was very scared about changing how I worked, [mindfulness] gave me the flexibility to try something different, so I did stuff like not working in the evenings, and not working in the mornings [before lectures] and I found that (a) I was happier, (b) I did better academically and (c) I had time to do other stuff with my life ...”* (Malpass et al. 2019).

Meditation helped in being more present to the patient, *“I feel calm and focused after I meditate which makes me more present for my patients. I am able to take a deep breath before entering a patient’s room and truly focus on that patient.”* (Weingartner et al. 2019).

Mindfulness increased compassion towards patients, and also enhanced the sense of connection with others, *“[I use] mindfulness when I am speaking with a patient or even with friends. When I start to zone out or get impatient with what they are saying I refocus and think to myself how important this is to them. And it makes me a kinder and more compassion doctor and friend.”* (Weingartner et al. 2019), *“My interpersonal relationships have improved since beginning mindfulness training.”* (Weingartner et al. 2019), *“I am more attentive, self-aware, curious and care more about my patients and myself as a result.”* (Garneau et al. 2013).

It also helped in coping with the difficulties of becoming a physician, *“…course allows us to take a deeper look into interpersonal patterns that are intrinsic to the profession and to find ways to cope with the difficulties of becoming a physician.”* (Garneau et al. 2013).

Participants reported how they used the learned skills and techniques when working in the hospital with deadlines (Moore et al. 2020) and towards the patients (Weingartner et al. 2019).

Better communication skills and mindful listening were considered important with patients, *“By enhancing my ability to examine the way I communicate with patients, I was able to recognize and correct certain errors I was making. For example, when I interviewed patients, I was quick, asking one question after another. Now I tend to use observant silence inviting the patient to say more.”* (Garneau et al. 2013).

**Factors affecting feasibility and satisfaction**

Participants were generally satisfied with mindfulness interventions. The timing of the course matters (Aherne et al. 2016), *“Bad timing of the course*”, *“I feel that Tuesday morning was our most stressful time and I would have been more receptive to the course had it been on another day”, “Being forced into assessing your stress while stressed is quite stressful”* (Aherne et al. 2016).

Content, amount and timing of meditations during the course may affect the feasibility (Aherne et al. 2016, Moore et al. 2020), *“Less discussion, more practice”, “I would have liked to do more practical exercises e.g. yoga, body scan and learnt more practical ways to practice mindfulness as opposed to how stressed I feel/ what stress is.”*(Aherne et al. 2016), *“more in-class practice”* (Greeson et al. 2015).

In one study comparing optional and mandatory mindfulness courses, students in the optional course were more satisfied with the course (Aherne et al., 2016). Mandatory course was not seen beneficial, *“Would only recommend if the person wanted to attend, being obligatory to attend meant that even if not open to the practice of mindfulness you still had to attend & I don't think you would get as much out if it as you would if it had it been optional.”* (Aherne et al. 2016), *“Some students in the group make it very difficult to relax, so I think there should be an alternative module for these people as it effects the overall experience.”* (Aherne et al. 2016). Some mandatory course students noted that mindfulness is not a technique that is suitable for everyone, *“Meditation isn't for everyone, alternate it with yoga and other stress relievers”* (Aherne et al. 2016).

Good and timely content of the course increases the feasibility, *“good content that increased the interest to continue”* (Moore et al. 2019), “*Introduce mindful walking, yoga, and S.T.O.P. as early as possible.”* (Aherne et al. 2016).

Teachers had significant role (Aherne et al. 2016, Weingartner et al. 2019), *“created a safe environment where his students could experience compassion training without judgment.”* *“His enthusiasm made it easy for me to move from a skeptical mindset towards acknowledgment of the benefits of meditation. [He] created a safe environment where his students could experience compassion training without judgment.”* (Weingartner et al. 2019)

The significance of peer group was evident, offering a safe, accepting and warm place to bond and develop (Malpass et al. (2019),*“The group discussions were helpful it was comforting to know other people had very similar thought patterns.*”(Malpass et al. 2019), *“I felt as a group there was a great bond and trust between us.”* (Aherne et al. 2016), *“I also liked having my class- mates around and knowing that I was not the only one feeling stressed.”* (Greeson et al. 2015).

**Difficulties and adverse effects**

Also some difficulties were reported. Noticing self-judgemental feelings was difficult and may cause anxiety. As Malpass et al. (2019) noted, “For some students, the course was the first time they saw clearly their self-judgemental thought patterns and felt their biggest obstacle was “their own mind, berating myself”. Some students find it difficult to sit still (Aherne et al. 2016), and it may be difficult to relax and sit still especially when stressed (Moore et al. 2020).

Adverse effects were not asked specifically, but some of the participants reported challenges and increased stress, *“I found alot of the course very challenging as it brought up and amplified a lot of thoughts that were very difficult for me to deal with. However it has helped me to look at them differently and although its still very hard”* (Malpass et al. 2019).

Mandatory mindfulness course increases stress *“If you have no capacity to relax, then this is very relevant - but if you can then this was almost stressful to keep on top of”* (Aherne et al. 2016).
